# Manipulating Majorana zero modes on atomic rings with an external magnetic field

**DOI:** 10.1038/ncomms10395

**Published:** 2016-01-21

**Authors:** Jian Li, Titus Neupert, B. Andrei Bernevig, Ali Yazdani

**Affiliations:** 1 Department of Physics, Princeton University, Princeton, New Jersey 08544, USA; 2 Princeton Center for Theoretical Science, Princeton University, Princeton, New Jersey 08544, USA

## Abstract

Non-Abelian quasiparticles have been predicted to exist in a variety of condensed matter systems. Their defining property is that an adiabatic braid between two of them results in a non-trivial change of the quantum state of the system. The simplest non-Abelian quasiparticles—the Majorana bound states—can occur in one-dimensional electronic nano-structures proximity-coupled to a bulk superconductor. Here we propose a set-up, based on chains of magnetic adatoms on the surface of a thin-film superconductor, in which the control over an externally applied magnetic field suffices to create and manipulate Majorana bound states. We consider specifically rings of adatoms and show that they allow for the creation, annihilation, adiabatic motion and braiding of pairs of Majorana bound states by varying the magnitude and orientation of the external magnetic field.

## Abstract (web summary)

Majorana fermions, particles which are their own antiparticles, are predicted to exist in systems combining superconductivity and topologically non-trivial band structure. Here, the authors propose means to create and manipulate such excitations in one-dimensional chains of adatoms on superconducting surfaces.

## Graphical abstract

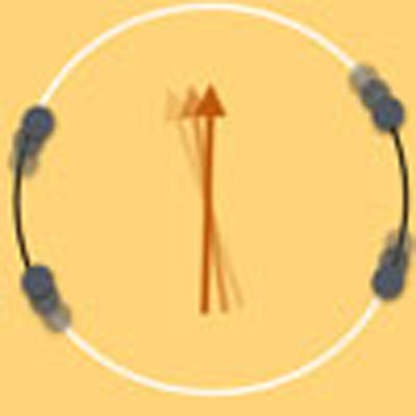

To date, no experimental platform has reached the desired control over non-Abelian quasiparticles to demonstrate their non-Abelian statistics. Quasi one-dimensional (1D) systems that have the potential to host Majorana bound states (MBS) are currently explored in great depth both theoretically^1^,^2^,^3^,^4^,^5^,^6^,^7^,^8^,^9^,^10^ and experimentally^11^,^12^,^13^,^14^,^15^,^16^. When tuned appropriately, such nano-wires can localize MBS at their ends, a pair of which forms a two-level system that is robust to local perturbations. This constitutes a topologically protected qubit that can serve as the building block for a topological quantum computer^17^,^18^,^19^,^20^,^21^. To implement braiding operations among the MBS, schemes that allow to move MBS across wire networks have been explored theoretically^8^,^22^.

One venue to physically realize a 1D topological superconductor that supports MBS are chains of localized magnetic moments, which are coupled to superconducting electrons^23^,^24^,^25^,^26^,^27^,^28^,^29^,^30^,^31^. Along the chain, Shiba bound states^32^,^33^,^34^,^35^,^36^,^37^ form in the superconducting gap. The superconducting electrons mediate Ruderman–Kittel–Kasuya–Yoshida (RKKY) interactions between the localized magnetic moments of the chain. This mechanism evokes a spiral magnetic order, where the pitch is given by either twice the Fermi momentum of the band of Shiba states if the RKKY interaction is 1D (ref. 38) or the Fermi surface of the underlying superconductor is largely 1D, or by the spin-orbit coupling if the RKKY interaction is two-dimensional (2D)^38^. In the anisotropic case, the back-action of the magnetic order on the conduction electrons lifts their degeneracy by opening a gap exactly at the Fermi energy for one spin orientation^23^,^39^,^40^,^41^,^42^. This effect occurs also in superconducting systems^26^, rendering them a topological 1D superconductor. In the more realistic 2D situation^38^, the existence and pitch of the helix depend on the amount of positional disorder and on the spin-orbit coupling interaction of the surface.

Helical magnetic chains have indeed been observed experimentally^43^. The mechanism responsible for the helix formation can be an interplay of various factors including RKKY interaction, Dzyaloshinskii–Moriya interaction and magnetocrystalline anisotropy. Here we show theoretically that such helical magnetic chains, formed on the surface of a thin-film superconductor, can be generically used to create and manipulate MBS by changing, respectively, the amplitude and orientation of an external magnetic field. Our conclusions hold independent of the specific mechanism responsible for the helix formation. Scanning tunnelling microscope (STM) tips can be used to control each magnetic atom's position individually to create any desired geometrical network of magnetic adatoms on the surface of a superconductor with the help of lateral atomic manipulation techniques^44^,^45^,^46^. STM can be further used to characterize the magnetic texture of these networks (using spin-polarized tips), as well as presence of MBS in a spatially resolved manner. In an applied external magnetic field of appropriately chosen strength, we find that a circular ad-atom chain features two trivial and two topological superconducting segments, whose interfaces each host a single MBS. The position of the MBS depends on the orientation of the external magnetic field in the plane of the superconducting surface. Rotating the magnetic field at an appropriate rate thus can adiabatically move the MBS along the circle. Based on this mechanism, we further propose structures that implement braiding and a *σ*_*x*_ gate operation on qubits formed from the MBS, via rotating the external magnetic field by 2*π*. All operations can be performed with the magnetic field in the plane of the superconducting surface, leaving thin-film superconductivity intact. We stress that, while the helix forming mechanism is currently under theoretical debate, the setups that we propose are not limited to systems with helical order. They can also be realized in Shiba-chains or nano-wires with Rashba spin-orbit coupling (SOC) and ferromagnetic order. The experimental realization of a ferromagnetic Shiba chain with MBS has been shown recently^16^.

## Results

### Magnetic structure of the chain

The magnetic order of the chain and its origin is a subject of intense scrutiny. The situation is likely to become even more theoretically complicated in the presence of an externally applied magnetic field which can possibly influence the formation and structure of the spin helix. In the absence of ab-initio calculations, we give a scenario of one of the possible outcomes for the chain helix, then, while assuming a generic helix configuration, look at the action of the magnetic field in a simple low-energy model which exposes the main idea of the paper. One of the potential magnetic orders in the 1D chain of magnetic moments on the (super)conducting surface is attributed to the RKKY mechanism. The interaction between the Fourier modes **S**_*q*_ of the spins 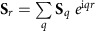 at sites *r*=1, ⋯, *L* of the chain is


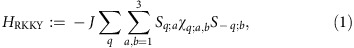


where *J*>0 is a coupling constant. In this scenario *χ*_*q*;*a*,*b*_ is the static magnetic susceptibility tensor of the (super)conducting electrons. There are differences between a 1D (which should be considered as the limit of anisotropic interactions) and 2D RKKY interaction: due to perfect nesting in 1D, the spin helix features a peak at twice the Fermi momentum *q*=2*k*_F_ due to resonant scattering between the Fermi points for interactions mediated by 1D normal^39^,^40^,^41^,^42^ or superconducting electrons^26^, while in the more realistic 2D case the interaction becomes short range and local ferromagnetic physics takes over^38^. In this latter case, intrinsic spin-orbit interaction can restore the helix. If the electronic structure is SU(2) spin-rotation symmetric, *χ*_*q*;*ab*_ is proportional to *δ*_*ab*_. As a consequence, the magnetic moments order in a helix structure 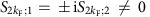. Due to the SU(2) invariance, any rotation of the helix in spin space is a degenerate symmetry breaking state. An arbitrarily small external magnetic field **B** breaks the SU(2) invariance and will pin the plane in which the helix rotates to be the plane normal to **B**.

This has to be contrasted to the situation where the magnetic chain is located on the surface of a superconductor (in the 1–2 plane, say). In this case the electrons mediating the (1D or 2D) RKKY interaction are subject to Rashba SOC due to the inversion-symmetry breaking of the surface. Along the 1D chain (the 1-direction, say), the Rashba SOC breaks the SU(2) spin-rotation symmetry down to U(1) spin-rotation symmetry in the 1–3 plane. This reduced symmetry is reflected by the spin susceptibility *χ*_*q*;*ab*_ being not diagonal anymore. Rather, it acquires a nonvanishing off-diagonal component *χ*_*q*;13_ and the diagonal components need not all be equal, but *χ*_*q*;11_=*χ*_*q*;33_≠*χ*_*q*;22_ (refs 38, 47, 48). An analysis of this for the simple but less realistic 1D RKKY case is presented in the Supplementary Note 1 and Supplementary Fig. 1. In particular, due to the different spin-polarizations of the Rashba-split Fermi points, the susceptibility peaks appear at different momenta in the components *χ*_*q*;11_=*χ*_*q*;33_ and *χ*_*q*;22_. As a consequence, the spin helix preferentially forms in the 1–3 plane, that is, 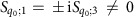 for some *q*_0_, while *S*_*q*;2_=0, ∀*q*. Further, the helix remains pinned to the 1–3 plane even in the presence of an external magnetic field **B** in the 1–2 plane, as long as |**B**| is smaller than some critical field *B*_c_. A simple estimate yields that *B*_*c*_ is given by the SOC-induced energy splitting of the the electronic bands that mediate the RKKY interaction at the Fermi level (see Supplementary Note 1 and Supplementary Figs 2–3). If |**B**| is much larger than *B*_c_, the helix forms in the plane normal to **B** (on top of an overall homogeneous magnetization in the direction of **B**).

In the remainder of this paper we will study the magnetically ordered chain with a magnetic field weaker than *B*_c_ in the 1–2 plane, so that the orientation of the helix is pinned to the 1–3 plane irrespective of the orientation of the external **B**. For simplicity, we shall *assume* that the magnetic helix is fully rigid and independent of **B**. For our purposes below, this is true as long as the energy scale *B*_c_ is much larger than the *p*-wave gap induced in the chain^24^. While this assumption is sufficient to demonstrate the key ideas of this proposal, a more complete analysis would have to include the influence of the magnetic field on the helical structure and the role of disorder. For example, a recent study indicates that strong disorder favors a ferromagnetic over the helical magnetic order^38^. We note, however, that the effects we exploit can also occur without the helical order, if instead a suitable combination of ferromagnetic order and Rashba SOC is realized; see the Supplementary Note 2 and Supplementary Figs 4–5 for details.

### Superconducting phases of a straight chain

We now want to study the effect of an external field **B** on the 1D chain of helical adatoms of length *L*. To that end, we consider the tight-binding Hamiltonian:


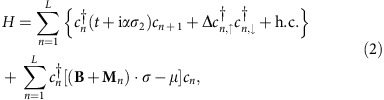


where 
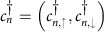
 and 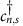 creates an electron of spin *s*=↑, ↓ on site *n*=1, ⋯, *L* and the lattice spacing is unity. Here, *t* is the nearest-neighbor hopping integral and Δ is the superconducting pairing potential. More realistic Hamiltonians for the chain involve obtaining hybridizations for the Shiba states^29^,^31^. Our Hamiltonian above is, however, simple enough to illustrate the point and also quantitatively accurate for chains larger than the underlying superconductor's coherence length. The magnetic moment of the helical order (assumed to lie in the 1–3 plane) has the spatial dependence **M**_*n*_=*M*[cos (*n θ*+*θ*_0_), 0,±sin (*n θ*+*θ*_0_)]^T^, where *M* is the overall amplitude, *θ* is the tilt between adjacent moments and ± stands for the two possible helicities. The overall phase *θ*_0_ has no significant effect on the spectrum of Hamiltonian (2) if the tilt angle *θ* is sufficiently small (see Supplementary Note 2 and Supplementary Fig. 6).

**Figure 1:**
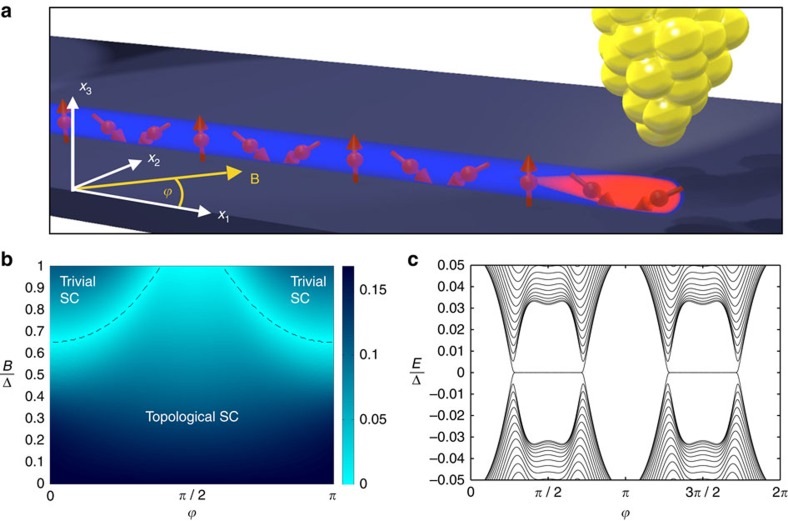
Properties of a straight Shiba chain. (**a**) The Shiba states (blue) of a chain of magnetically ordered adatoms (red) on a thin-film superconductor are a one-dimensional topological superconductor that features a Majorana end state (orange). A scanning tunneling microscope (STM) tip (yellow) can be used to detect it as a zero-bias anomaly of the tunneling current. (**b**) Phase diagram of Hamiltonian (2) in terms of the magnitude *B* and the angle 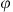 (with respect to the 1-direction) of the external field **B**. The colour scale indicates the ratio between the induced gap Δ_necklace_ and the original superconducting gap Δ. The parameter values are *μ*/Δ=1.5, *M*/Δ=3.4, *α*/Δ=0.75, *t*/Δ=1 and *θ*=*π*/6. At constant |**B**|, topological phase transitions are possible as a function of 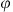. (**c**) Spectrum of the finite chain of *L*=600 sites as a function of 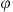 for *B*/Δ=0.8 and otherwise the same parameters. The straight lines at zero energy are Majorana zero energy states bound to the end of the chain.

If 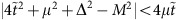, with 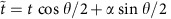, and the external magnetic field **B** vanishes, the ground state of Hamiltonian (2) is a topological 1D superconductor with MBS at its end. We now propose to use magnetic fields in the 1- or 2-direction to drive the system into a topologically trivial state without MBS at its end. Upon increasing *B*_2_ (with *B*_1_=0), the system either enters a gapless phase at the critical field strength *B*_2,c_=Δ (if 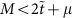) or enters a gapped but topologically trivial phase at 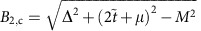 (if 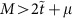). Similarly, yet by a very different mechanism, increasing *B*_1_ (with *B*_2_=0) can trigger the transition into a topologically trivial gapped phase at some critical field strength *B*_1,c_. Unlike with *B*_2,c_, a closed analytical expression for *B*_1,c_ can only be obtained in the limit of small Δ and *B*_1_ (see Supplementary Note 2). Crucially, the critical field strengths at which the transition occurs is generically different for fields in the 1- and 2-direction, and for appropriate choices of parameters *B*_1,c_<Δ (see Supplementary Note 2). For example, if *B*_1,c_<*B*_2,c_ and the magnitude *B* of the external field **B** is chosen such that *B*_1,c_<*B*<*B*_2,c_, a phase transition between the topological and trivial superconducting state of the chain can be crossed by *rotating* **B** in the 1–2 plane across a critical angle 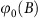, keeping its magnitude *B* fixed (Fig. 1c). This topological phase transition as a function of the orientation of **B** in the 1–2 plane is the key property of the Shiba chain that we will exploit in the remainder of this work. It should be noted that the model parameters need to satisfy the following two conditions: *B*_1,c_<*B*<*B*_c_ in order to pin the magnetic structure; sufficiently large gaps for **B** in the 1- and 2-directions to protect the coherent manipulations considered below. For the parameters chosen in Fig. 1c, *B*/Δ=0.8<1.0≃*B*_c_/Δ. If the Rashba SOC is larger, this inequality can be made more strong, for example, for *α*/Δ=5.0, *M*/Δ=4.8, and *μ*/Δ=1.0, a phase diagram similar to Fig. 1c is obtained while 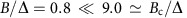.

### The necklace

**Figure 2:**
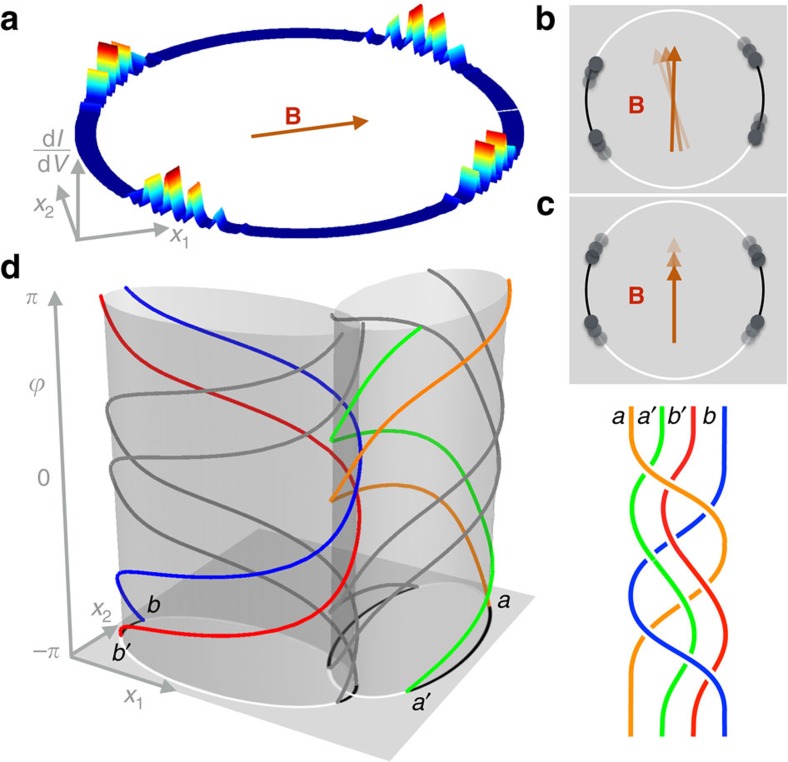
Properties of a circular Shiba chain and a *σ*_*x*_ gate. (**a**) For appropriate external magnetic field strength |**B**|, the circular magnetic chain (*L*=600 in this example) on a thin-film superconductor hosts four Majorana bound states (MBS) that can be measured as zero-bias anomalies with an STM tip. (**b**) Changing the orientation of **B** moves the MBS along the circle. (Black are trivial, white are topological sectors of the chain.) (**c**) Changing the magnitude of **B** controls their distance and allows to create and fuse them pairwise. (**d**) Two overlapping elliptic chains can be used as a *σ*_*x*_ gate that is operated by a 2*π* rotation of the angle 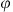 of **B** in the 1–2 plane. Shown are the world lines of the MBS during the braid. Observe that the orange MBS braids around the red, green and blue MBS, while the green MBS braids around the blue and orange MBS only.

To to move Majorana bound states in a controlled way, they have to appear at some mobile domain wall between a trivial and a topological superconducting state. Given the dependence of the topological phase transition on the orientation of the external magnetic field, such domain walls can occur between chain segments with different relative orientations to **B**. One can join up many such segments into a bent chain or a circle. For the latter, the angle 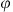 between the homogeneous external field **B** and (the tangential to) the circular chain becomes position dependent as 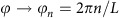. If 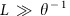, the local magnetic structure of the chain will not be affected by the bending. Locally, the helical magnetic moment is taken to lie in the plane spanned by the tangential to the circle and the 3-direction. We choose the length *L* such that no frustration or magnetic domain wall are induced due to the periodic boundary conditions, that is, 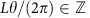. Then, if *B*_1,c_<*B*<*B*_2,c_, two segments of the circle with angles 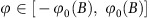 and 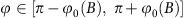 are trivial while the two segments separating them are topological 1D superconductors. Each of the four domain walls between these segments host a single MBS (Fig 2a). For any finite *L*, the finite size splitting of the MBS due to their hybridization is exponentially small in *L*/*ξ*, with *ξ* the coherence length. Note that the energy separation of the MBS from excited states also scales to zero in the thermodynamic limit *L*→∞, because the circle and with it also the shape of the domain wall are self-similar for different *L*. The situation is similar to that of a very soft boundary between topological and nontopological phases. However, the excitation energy only scales to zero as *ξ*/*L* or slower (Supplementary Note 2 and Supplementary Fig. 7–10). For a finite circle, this weak scaling leaves several orders of magnitude in energy between the MBS splitting and the lowest excitation energy. For example, with the parameters used in Fig. 1b and *L*=600, the MBS splitting is 10^−5^Δ while the energy of the first excited state is 0.035Δ. On the intermediate energy (or time) scales, we now can perform dynamical operations on the MBS. For example, the four MBS on the circle can be moved along the circle by rotating the magnetic field, while maintaining their relative positions (Fig. 2b).

### The *σ*_*x*_ gate

The control over magnitude and angle of the external magnetic field is sufficient to create and annihilate qubits of MBS and to perform gate operations on the qubits, as we now show. Two distant MBS *a* and *a*′ form a nonlocal fermionic two-level system with occupation eigenstates |0〉_*a*_ and |1〉_*a*_ for zero and one fermion, respectively. All topologically protected adiabatic operations, such as the rotation of the external field, do not change the fermion parity of the superconducting chain. Hence, qubit operations have to be performed on a Hilbert space of constant parity. For that reason, we consider a qubit made of two pairs *a*, *a*′ and *b*, *b*′ of MBS. Specifically, we consider a set-up of two intersecting ellipses as sketched in Fig. 2d, where the MBS pair *a*, *a*′ is located on one ellipse, while the pair *b*, *b*′ is on the other. Figure 2d shows the world lines of the MBS under a 2*π* rotation of the external field **B**. This process braids the MBS *a* with *a*′, *b*, and *b*′, while it braids *a*′ with *a* and *b*, but not with *b*′. As a result, the qubit formed from *a*, *a*′, *b*, and *b*′ changes as


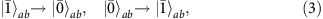


where we denoted the qubit eigenstates as 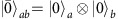 and 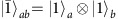. This constitutes a *σ*_*x*_ gate operation. Each ellipse in Fig. 2d hosts a second pair of MBS, whose world lines are indicated in grey. These MBS do not interfere with the qubit operation on *a*, *a*′ and *b*, *b*′. Rather, they form a second qubit on which the same operation is performed. Up to a global phase, the transformation (3) depends only on the topology of the world lines of the MBS and not on any details of the adiabatic evolution. We have confirmed equation (3) via an explicit calculation of the adiabatic evolution of the many-body states under the field rotation using a simplified model (Supplementary Note 3, Supplementary Figs 11–14 and Supplementary Table 1–3).

### The braiding operation

**Figure 3:**
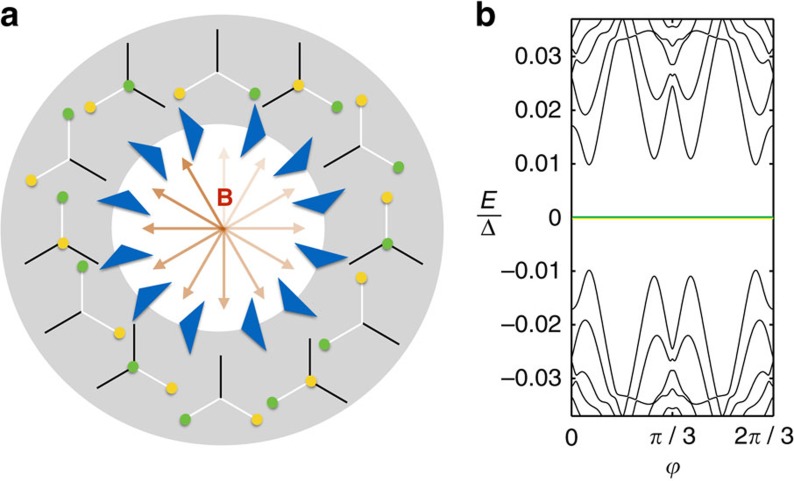
Realization of a phase gate. (**a**) A trijunction of chains in an inplane magnetic field *B* supports two MBS and can be used to braid two MBS via a 2*π* rotation of **B**. (**b**) Spectrum of the trijunction as a function of the orientation 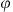 of **B**. The spectrum is 2*π*/3-periodic. Each chain of length *L*=180 is governed by Hamiltonian (2) with the parameter values *μ*/Δ=1.5, *M*/Δ=3.4, *α*/Δ=0.75, *t*/Δ=1, and *θ*=*π*/6.

We now focus on a qubit composed of a single pair of MBS *a* and *a*′ and want to perform a braiding of the two MBS. This operation conserves the parity of the qubit. We use a trijunction of straight chains to demonstrate the operation (Supplementary Note 3 and Supplementary Fig. 15). When choosing 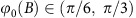, the trijunction hosts two MBS with one or two topological segments for every inplane orientation of **B**. As sketched in Fig. 3a, a rotation of **B** by 2*π* braids the two MBS around one another. In this process, the states of the qubit transform as |0〉_*a*_→|0〉_*a*_ and |1〉_*a*_→−|1〉_*a*_, modulo a nonuniversal overall U(1) phase factor multiplying both basis states. Figure 3b shows the spectrum of the trijunction during the braid. It should be noted that the gap that protects the MBS collapses for certain orientations of **B** as the number of sites in each segment is taken to infinity. However, the finite size gap can be several orders of magnitude larger than the splitting between MBS. (The gaps are 0.01Δ and 10^−6^Δ, respectively, for the parameter values studied in Fig. 3b.)

### Measurement and experimental implementation

We propose local experimental probes, namely an STM and a scanning single-electron transistor (SET) to demonstrate that the MBS exist, can be moved and can be braided in the proposed necklace set-up. To show the existence of MBS, STM probes zero-bias anomalies (ZBA) at four positions along the circle. While open wires might host nontopological end states that can be confused with the MBS, ZBAs in the necklace directly evidence the magnetic field induced topological phase transition. To show the movement of MBS, STM measurements at different orientations of inplane magnetic field, will find the MBS at different locations along the chain. As long as *B*<*B*_1,c_, STM measurements will show no ZBA at any location along the necklace. For *B*_2,c_>*B*>*B*_1,c_, four ZBAs will appear in every rotation period of **B**. As these measurements are sensitive to the density of states of the MBS, but not their quantum state, they do not suffer from decoherence processes. In contrast, to show the braiding of MBS, the quantum state of the MBS has to be prepared before the gate operation and measured thereafter. We propose the following protocol using the set-up of Fig. 2. First, *B* is increased adiabatically beyond *B*_1,c_, at which point pairs of MBS are created. Due to the finite overlap of the MBS, each pair's eigenstates |0〉 and |1〉 are split in energy and each is prepared in the lower state |0〉. Second, for appropriate *B*_2,c_>*B*>*B*_1,c_, the *σ*_*x*_ gate operation is implemented by a 2*π* rotation of **B**. This changes the state of each of the four pairs of MBS to |1〉, taking two Cooper pairs from the condensate. Third, the MBS are fused pairwise by reducing the magnetic field *B*→*B*_1,c_. Integration of a scanning SET within an STM step-up will be required to probe the local charge density and sense the presence (or absence) of remnant charge and state of the Majorana qubits after manipulation. It is important not to use the STM tunnelling at all during such manipulation, as tunnelling of quasiparticles from an STM tip will cause decoherence. A key advantage of our proposed approach is that metal contacts or gates (that are sources of unpaired poisoning quasiparticles) are not required for manipulation of MBS.

## Discussion

We close with some crude estimates of the energy and time scales relevant to the experimental realization of our braiding proposal. The detection of MBS through the STM measurement can be made in a slowly rotating magnetic field and hence is not subject to the more strict requirements necessary for braiding. Quasiparticle poisoning can be as hazardous to this as to other platforms that realize MBS^22^,^49^,^50^,^51^,^52^. It reduces the height of the ZBA and is believed to be the main source of decoherence during a braiding operation. Typically, the thin-film superconducting gap Δ∼1 meV, so that the gap protecting the MBS in the straight chain or necklace Δ_necklace_∼0.05 meV. We can estimate thermal quasiparticles to appear at a rate *ω*_qp_∼0.001 meV (which is much larger than the finite size MBS splitting). This will, however, require significant efforts in cooling the system.

Another challenge is to perform the field rotation of the field *B*∼0.5 T at a frequency *ω* that is quasi adiabatic wrt. Δ_necklace_ but large wrt. *ω*_qp_, that is, *ω*∼0.01 meV∼2 GHz. A set-up in which current pulses running through two perpendicular superconducting wires create a rotating magnetic field can be envisioned (Supplementary Note 3 and Supplementary Fig. 16). The viability of our proposal thus depends sensitively on the ability to generate a system with *ω*_qp_ and Δ_necklace_ in the range obtained with our model calculations, as magnetic field rotations at even higher frequencies would be hard to realize. Furthermore, in an implementation of our proposal, one needs to consider the back-reaction of the rotating field on the magnetic structure of the chain and the associated heating effects.

In summary, using the Shiba states of magnetic adatoms on the surface of a thin-film superconductor, we have proposed a system that allows for the detection and manipulation of MBS and in particular can be used to demonstrate their non-Abelian character. Our proposal takes full advantage of the high level of control that one has regarding the design, operation and measurements using STM in this set-up.

## Methods

### Theoretical calculations and modelling

To deduce the magnetic order of the chain, an RKKY calculation and a subsequent minimization of the classical magnetic system was performed. The electronic structure was analysed by exact diagonalization of tight-binding Bogoliubov-de Gennes Hamiltonians based on equation (2) for the various structures considered. To simulate the braiding operation under the rotation of an external magnetic field, we used a simplified model based on a 2D spinless p-wave superconductor, defined in Supplementary Note 3 and Supplementary Fig. 13, and numerically calculated the overall many-body Berry phase acquired by the ground states, together with the monodromy braiding matrix, by using the Wilson-loop method.

## Additional information

**How to cite this article:** Li, J. *et al.* Manipulating Majorana zero modes on atomic rings with an external magnetic field. *Nat. Commun.* 7:10395 doi: 10.1038/ncomms10395 (2016).

## Supplementary Material

Supplementary Information Supplementary Figures 1-16, Supplementary Tables 1-3, Supplementary Notes 1-3 and Supplementary References
