# Supplementary material for: Manipulating Majorana zero modes on atomic rings with an external magnetic field
Source: Nat Commun. 2016 Jan 21;7:10395. doi: 10.1038/ncomms10395 (PMC4735899; doi:10.1038/ncomms10395)
Supplement: Supplementary Information — Supplementary Figures 1-16, Supplementary Tables 1-3, Supplementary Notes 1-3 and Supplementary References [file ncomms10395-s1.pdf]

Supplementary Figures

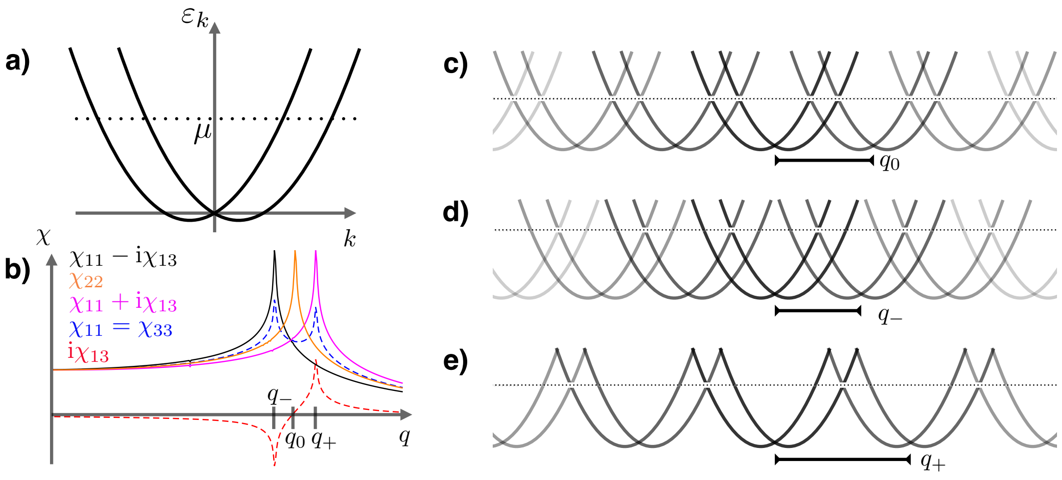

Supplementary Figure 1: a) Schematic band structure of the Rashba Hamiltonian (1). b) Various components of the susceptibility tensor for the non-superconducting state with Rashba spin-orbit coupling. The single peak or pair of peaks appear in the components χ_{22} and $\chi_{11} = \chi_{33}$, respectively, where the splitting between the peaks is determined by the spin-orbit coupling length. c)-e) Band hybridization caused by the three magnetic instabilities (spin-density wave and two helical spin states with opposite helicity) at the ordering wave vectors q_0 and $q_\pm$.

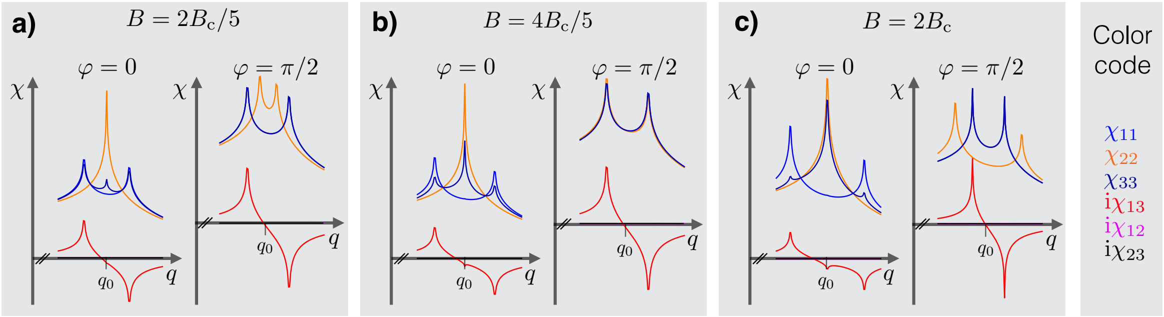

Supplementary Figure 2: Various components of the susceptibility tensor for the non-superconducting state with Rashba spin-orbit coupling and an external magnetic field according to Eq. (23). (a) $B = 0.2$, (b) $B = 0.4$, which is close to B_c defined in Eq. (25), and (c) $B = 1.0$. The curves for $i\chi_{12}$ and $i\chi_{23}$ are degenerate along the horizontal axis in all panels. The curves for χ_{11} and χ_{33} are degenerate for $\varphi = \pi/2$ in all panels. The momentum q_0 is defined below Eq. (8).

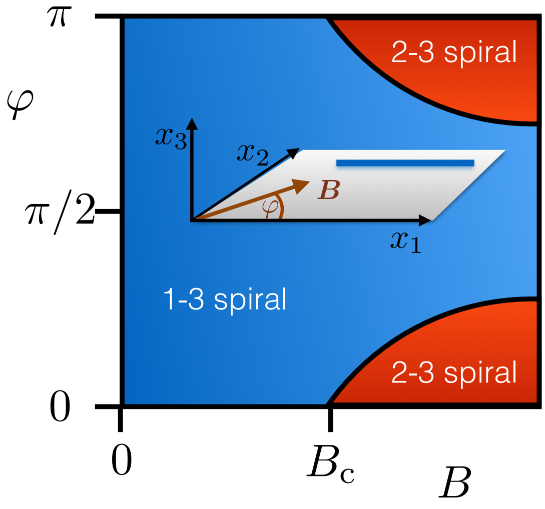

Supplementary Figure 3: Schematic phase diagram for the influence of a homogeneous magnetic field on the helical magnetic order. If $B \ll B_c$ a helix in the 1-3 plane is formed independent of the field orientation φ. For large magnetic field, in contrast, we observe a tendency for forming a helix in the plane perpendicular to the magnetic field. This phase diagram only shows the effect of the homogeneous magnetic field on the spatially varying (helical) component of the magnetization. In addition to the modification of the helical order, the homogeneous magnetic field will also induce an overall homogeneous magnetization parallel to the field, which will grow with B.

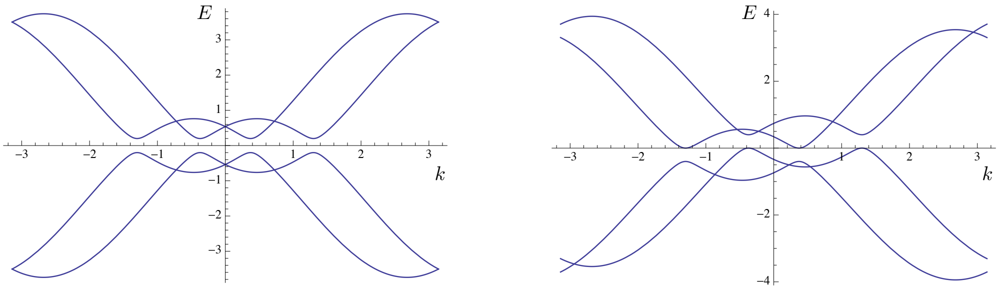

Supplementary Figure 4: Spectrum of Hamiltonian (26) for $\mu/t = 1.5$, $\Delta/t = 0.2$, $\mu/t = 0.5$, as well as $\mathbf{B} = 0$ (left panel) and $\mathbf{B} = (0, 0.2, 0)$ (right panel). For the parameter values on the right panel, i.e., $|B_2| = |\Delta|$, the (indirect) superconducting gap closes.

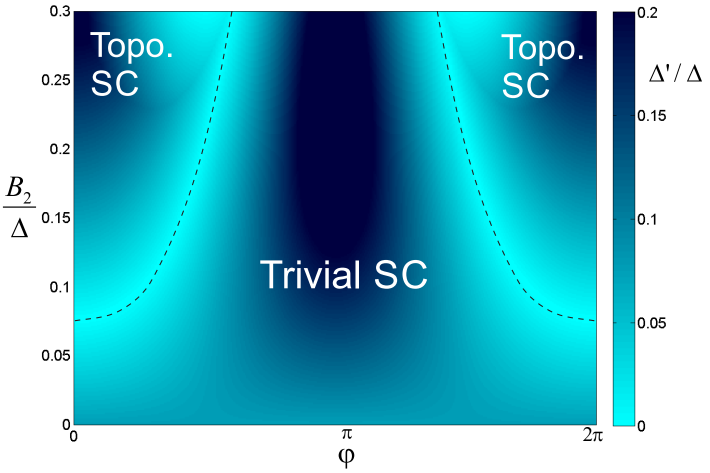

Supplementary Figure 5: Phase diagram of a Rashba wire in a combined magnetic field $\mathbf{B} = (B_1 + B_2 \cos\varphi, B_2 \sin\varphi, 0)$ with $B_1 = 0.24$. The other parameters are $t = -1$, $\mu = -1.9$, $\alpha = 0.4$ and $\Delta = 0.3$. Note in particular that here φ varies from 0 to 2π whereas in Figure 1(b) of the main text φ varies from 0 to π.

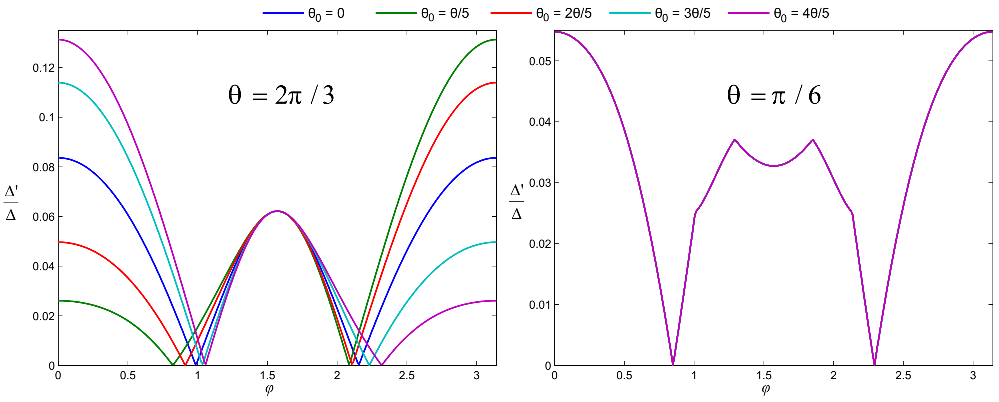

Supplementary Figure 6: Effects of θ_0 on the bulk band gap, shown as a function of the orientation of the external $\mathbf{B}$-field with fixed $|\mathbf{B}|$ (cf. Figure 1 of the main text). For large θ (left panel, $\theta = 2\pi/3$), the dependence of the spectra on θ_0 is significant; for small θ (right panel, $\theta = \pi/6$), the dependence is almost indiscernible (all curves here fall on top of each other). The parameters used for the left panel are $\mu/\Delta = 3.9$, $M/\Delta = 5$, $\alpha/\Delta = 0.75$, $t/\Delta = 1$, $|\mathbf{B}|/\Delta = 0.65$; the parameters used for the right panel are the same as those for Figure 1(c) of the main text.

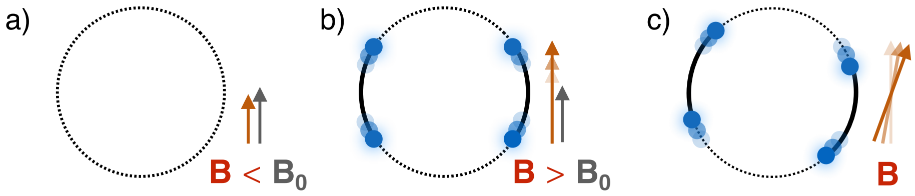

Supplementary Figure 7: Majorana zero energy bound states on a circle. (a) If the external magnetic field B is below a threshold value $B_{1,\mathrm{c}}$ the entire circle is in a topological superconducting state. (b) Once B is in between $B_{1,\mathrm{c}}$ and $B_{2,\mathrm{c}}$, two trivial superconducting segments open up at opposite end of the circle. Each interface between a trivial and a topological segment hosts a Majorana bound state (blue dot). (c) The position of the bound states depends on the field orientation. The Majorana zero energy modes can thus be moved by rotating the external magnetic field. Notice that each of the 4 Majorana states braids once around each other Majorana state during a 2π rotation of the external magnetic field.

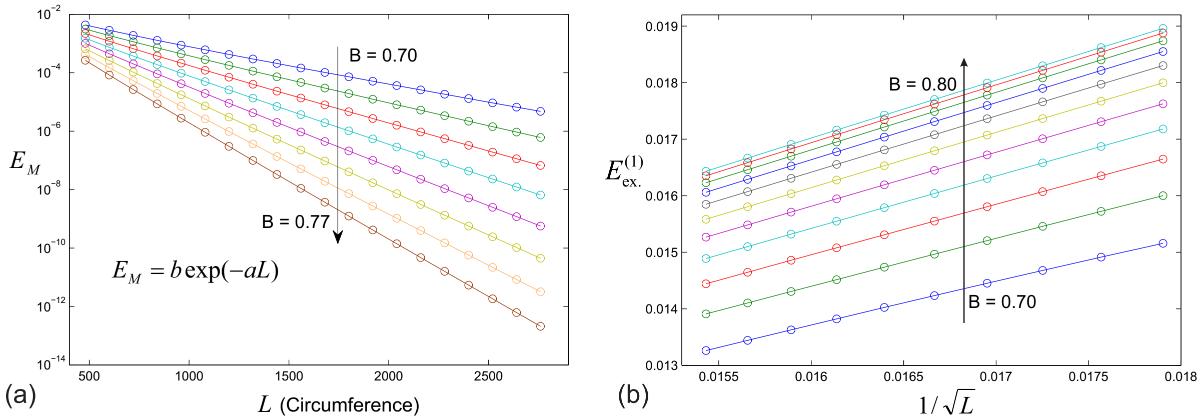

Supplementary Figure 8: Finite size scaling of the low-energy domain wall states in the circle geometry. (a) The energy-splitting of the Majorana bound states scales exponentially in L. (b) The energy of the first excited state above the Majorana bound states scales as $1/\sqrt{L}$. Cases are compared with varying external magnetic field B and otherwise the same parameters as in Figure 2 of the main text.

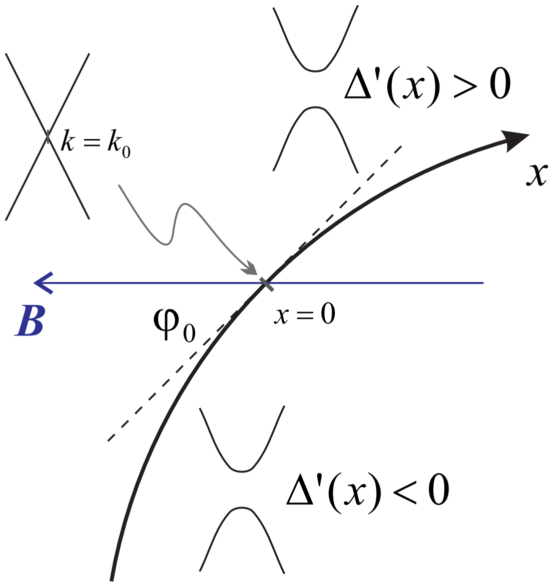

Supplementary Figure 9: Illustration of the effective model Eq. (58) describing a domain wall on a circle.

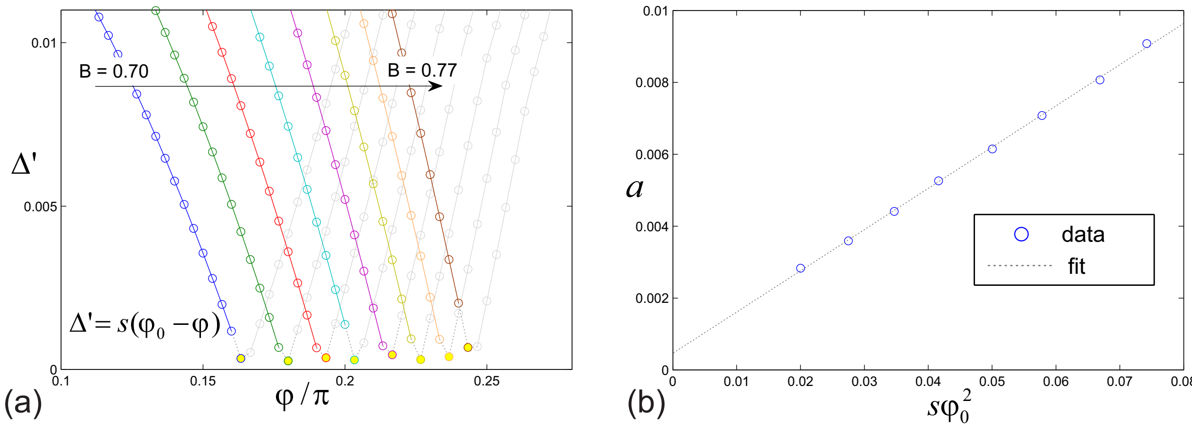

Supplementary Figure 10: (a) Bulk band gaps Δ' in the vicinity of topological phase transitions. (b) Test of the correlation predicted by Eq. (62).

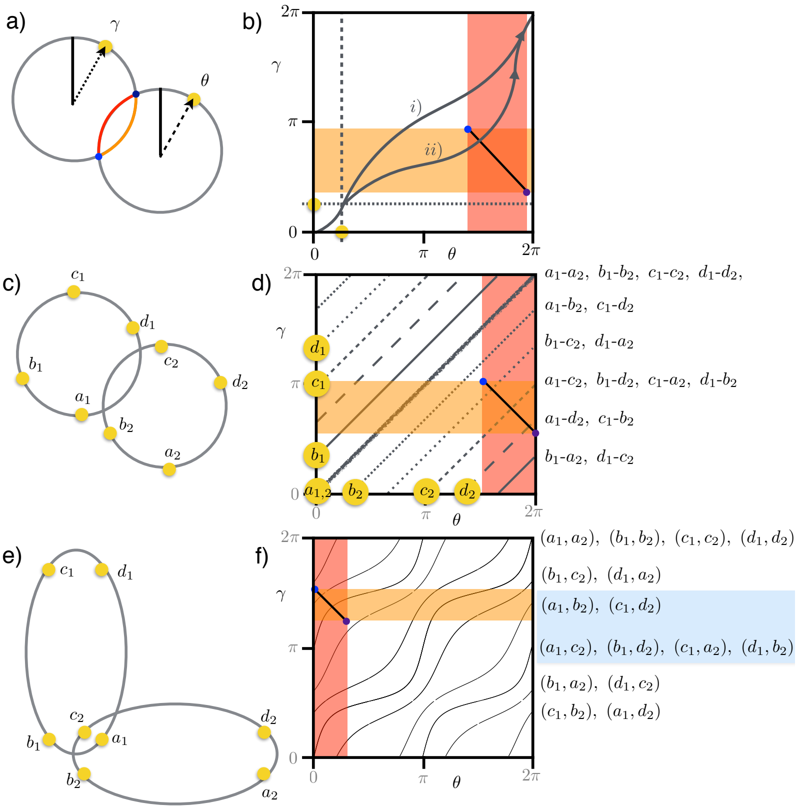

Supplementary Figure 11: Implementation of a σ_x gate with two elliptic chains via a 2π rotation of the magnetic field. a) Two circles or ellipses are parametrized by an angle θ and γ. Their relative position is fixed by two intersection points (blue dots) and the information which segment of each circle is encircled by the other one (orange/red lines). b) γ-θ-plane for the (unphysical) case of one Majorana on each circle (yellow dots). As the $\mathbf{B}$-field rotates, the Majoranas move along a trajectory in this plane (grey lines). If the trajectory traverses the black line connecting the two blue intersection points of the circles, the Majoranas are braided upon a 2π rotation of $\mathbf{B}$ [case ii)]. Otherwise, the Majoranas are not braiding [case i)]. c) The physical case of four Majoranas on each circle. d) Circles cannot be used to implement a σ_x gate operation, for each Majorana fermion experiences an even number of braiding operations due to the high symmetry. This can be seen from the fact that two times an even number of trajectories intersect the black line, according to the discussion in the text. Notice that the short-dashed trajectory is 4-fold degenerate, while the dotted and the long-dahed trajectories are 2-fold degenerate, according to the Majorana pairs listed next to the trajectories. Hence, a total of $4 + 2 \times 2 = 2 \times 4$ trajectories intersect the black line. e) An arrangement of two ellipses that can be used to realize a σ_x gate operation. f) The pairs of Majoranas marked in blue are braided for this configuration of the ellipses. Again, notice that on of the trajectories that cross the black line is 4-fold degenerate and the other one is 2-fold degenerate, so that a total of $4+2 = 2\times 3$ trajectories cross.

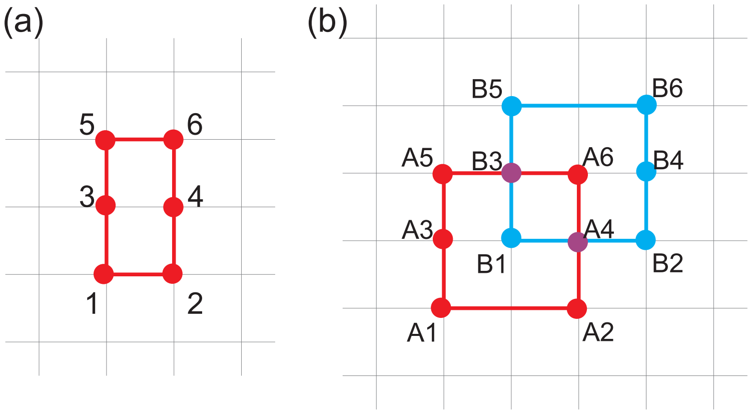

Supplementary Figure 12: Minimal lattice models to simulate Majorana braiding operations in (a) a single ring and (b) two linked rings. These models are constructed from a two-dimensional lattice model for spinless p-wave superconductors. The construction is to pick out a small number of lattice sites and bonds (highlighted) that fulfill the intended structure. Note that in (b) we treat sites $A1$ and $A2$, as well as $B5$ and $B6$, as nearest neighbors in the original (2D) lattice.

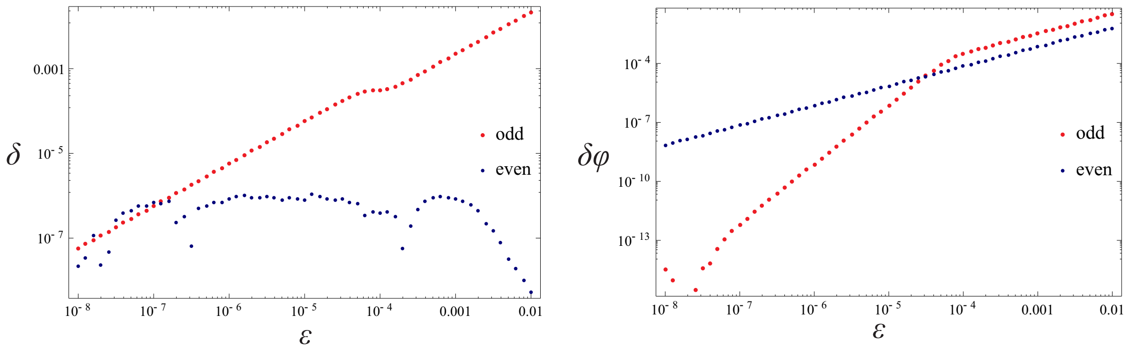

Supplementary Figure 13: Errors for the operation considered in the single-ring case, as the coupling ϵ among Majorana states is increased. δ stands for magnitude of the off-diagonal elements in the braiding matrices (for the odd and the even parity sectors separately), and $\delta\varphi$ stands for the phase error in the diagonal elements.

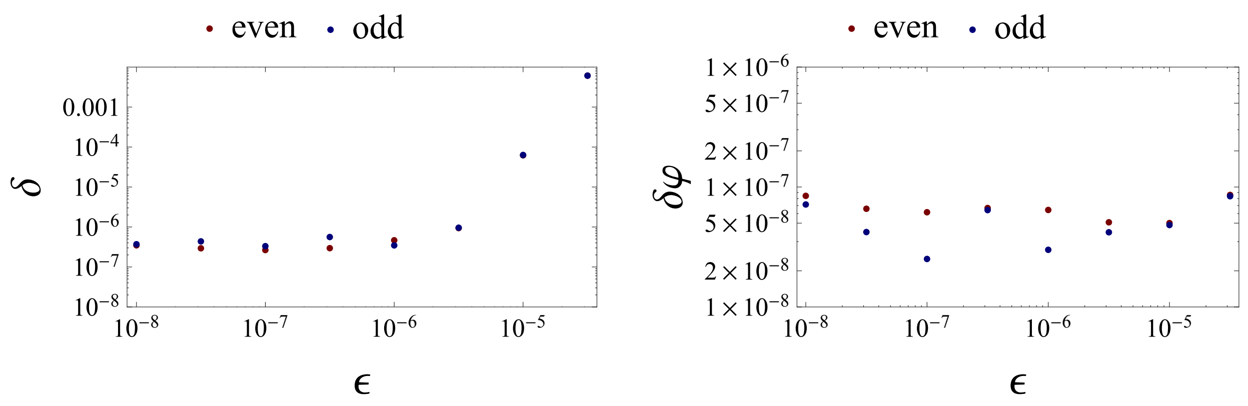

Supplementary Figure 14: Errors for the operation considered in the linked-ring case, separately for the even and the odd sectors, as the coupling ϵ among Majorana states is increased. δ stands for the probability error, and $\delta\varphi$ stands for the phase error (see text for their definitions).

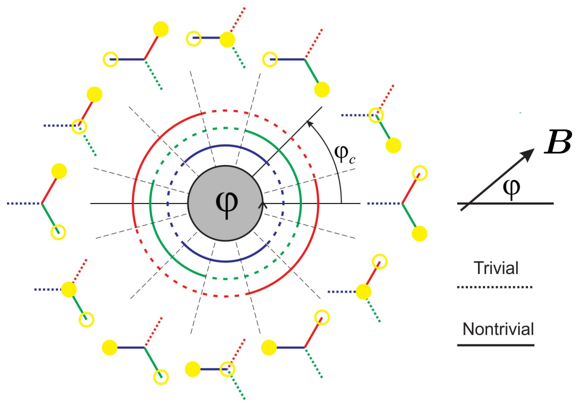

Supplementary Figure 15: Implementation of the braiding operation on a qubit formed by a single pair of Majorana states in a trijunction of chains by a 2π rotation of the external magnetic field $\mathbf{B}$.

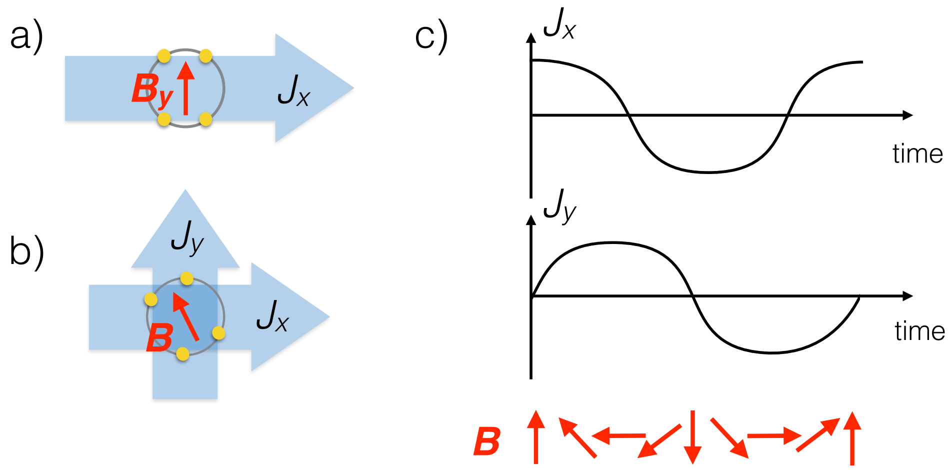

Supplementary Figure 16: Possible setup for fast rotation of the magnetic field in the Majorana necklace using current pulses. (a) A conductor (blue) is placed below the thin superconducting film with the chain of adatoms on top. A current J_x running through the conductor produces a magnetic field component B_y perpendicular to the current at the location of the necklace, above the conductor. (Notice that the thin superconductor cannot screen magnetic fields which are in the plane of the thin film.) (b) Two perpendicular conductors with tunable currents J_x and J_y can be used to produce a magnetic field of arbitrary in-plane orientation at the position of the necklace. (c) Sinusoidal current pulses with a $\pi/2$ phase shift between J_x and J_y create a rotating magnetic field at the position of the necklace.

Supplementary Tables

	a_1	b_1	c_1	d_1	a_2	b_2	c_2	d_2	Parity of braidings
a_1	0	1	1	1	0	1	1	0	-1
b_1	1	0	1	1	0	0	0	1	+1
c_1	1	1	0	1	1	0	0	1	-1
d_1	1	1	1	0	0	1	0	0	+1
a_2	0	0	1	0	0	1	1	1	+1
b_2	1	0	0	1	1	0	1	1	-1
c_2	1	0	0	0	1	1	0	1	+1
d_2	0	1	1	0	1	1	1	0	-1

Supplementary Table 1: The number of braidings between each pair of Majoranas, as well as the parity of the total braiding operation on each Majorana, upon a 2π rotation of the magnetic field in the case of $p = 2q$.

nodes	non-zero parameters
0	$\mu_3, \mu_4, t_{1x}, t_{5x}$
1	$t_{1x}, t_{5x}, t_{2y}, t_{3y}$
2	$\mu_1, \mu_2, \mu_5, \mu_6, t_{1x}, t_{5x}$
3	$t_{1x}, t_{5x}, t_{1y}, t_{4y}$
4	same as 0

Supplementary Table 2: Parameter settings at intermediate stages in an exchange operation of four Majoranas on a single ring. We only list the non-zero parameters, which are all of value 1 at the these stage. The full parameter trajectory can be obtained by linearly interpolating values between consecutive stages.

nodes	non-zero parameters
0	$\mu_{A3}, \mu_{A4}, \mu_{B3}, \mu_{B4}, t_{A1x}, t_{A4x}, t_{A5x}, t_{B1x}, t_{B3x}, t_{B5x}$
1	$\mu_{A4}, \mu_{B3}, t_{A1x}, t_{A4x}, t_{A5x}, t_{B1x}, t_{B3x}, t_{B5x}, t_{A3y}, t_{B2y}$
2	$\mu_{A4}, \mu_{A5}, \mu_{A6}, \mu_{B1}, \mu_{B2}, \mu_{B3}, t_{A1x}, t_{A4x}, t_{A5x}, t_{B1x}, t_{B3x}, t_{B5x}$
3	$\mu_{A4}, \mu_{A5}, \mu_{A6}, \mu_{B1}, \mu_{B3}, t_{A1x}, t_{A4x}, t_{A5x}, t_{B1x}, t_{B3x}, t_{B5x}, t_{A4y}, t_{B1y}, t_{B3y}$
4	$\mu_{A4}, \mu_{A6}, \mu_{B1}, \mu_{B3}, t_{A1x}, t_{A4x}, t_{A5x}, t_{B1x}, t_{B3x}, t_{B5x}, t_{A2y}, t_{A4y}, t_{B1y}, t_{B3y}$
5	$\mu_{A4}, \mu_{A6}, \mu_{B1}, \mu_{B3}, \mu_{B5}, \mu_{B6}, t_{A1x}, t_{A4x}, t_{B1x}, t_{B3x}, t_{B5x}, t_{A2y}, t_{A4y}, t_{B1y}$
6	$\mu_{A1}, \mu_{A2}, \mu_{A4}, \mu_{A6}, \mu_{B1}, \mu_{B3}, \mu_{B5}, \mu_{B6}, t_{A1x}, t_{B1x}, t_{B3x}, t_{B5x}, t_{A4y}, t_{B1y}$
7	$\mu_{A1}, \mu_{A2}, \mu_{A4}, \mu_{A6}, \mu_{B3}, \mu_{B5}, \mu_{B6}, t_{A1x}, t_{A5x}, t_{B1x}, t_{B3x}, t_{B5x}, t_{A4y}$
8	$\mu_{A1}, \mu_{A2}, \mu_{A4}, \mu_{B3}, \mu_{B5}, \mu_{B6}, t_{A1x}, t_{A4x}, t_{A5x}, t_{B1x}, t_{B3x}, t_{B5x}$
9	$\mu_{A1}, \mu_{A4}, \mu_{B3}, \mu_{B6}, t_{A1x}, t_{A4x}, t_{A5x}, t_{B1x}, t_{B3x}, t_{B5x}, t_{A1y}, t_{B4y}$
$10 \to 19$	repeat $0 \to 9$
20	same as 0

Supplementary Table 3: Parameter settings at intermediate stages in a braiding operation of Majoranas in the linked-ring case. We only list the non-zero parameters with their values set to 1 at the these stages. The full parameter trajectory can be obtained by linearly interpolating values between consecutive stages.

Supplementary Note 1. Magnetic structure of a straight chain

We consider the classical magnetic order that emerges due to 1D RKKY interaction mediated by electrons that are subject to an extra Rashba spin-orbit coupling. The Rashba spin-orbit coupling induces Dschaloschinskii-Moriya interactions between the spins, resulting in a helical magnetic order of preferred orientation. The effect of an externally applied Zeeman field is studied. If large enough, the field can change the direction in which the helix forms. We stress that these calculations are valid only for pure 1D RKKY interaction, which is not the realistic situation in a 2D sample. However, it is simple enough to be solved analytically, and gives a hint of the effects of an external magnetic field action.

A. RKKY interactions in presence of Rashba spin-orbit coupling

Consider electrons of effective mass m that are confined in a one-dimensional channel and governed by the second-quantized Hamiltonian

$$H_0 := \sum_k c_k^\dagger \left(\frac{k^2}{2m} - \mu + \mathbf{g}_k \cdot \boldsymbol{\sigma} \right) c_k, \tag{1}$$

where $c_k = (c_{k,\uparrow}, c_{k,\downarrow})^\mathsf{T}$, and $c_{k,s}^\dagger$ creates an electron of momentum k and spin $s =\uparrow, \downarrow$. The vector $\mathbf{g}_k = -\mathbf{g}_{-k}$ parametrizes the spin-orbit coupling in the system and $\boldsymbol{\sigma} = (\sigma_1, \sigma_2, \sigma_3)$ is the vector of the three Pauli matrices acting in spin space. The spectrum of H_0 consists of two spin-orbit split branches and is given by

$$\xi_{k,\lambda} := \frac{k^2}{2m} - \mu + \lambda |\mathbf{g}_k|, \qquad \lambda = \pm. \tag{2}$$

The conduction electrons couple via the Hund's coupling of strength β

$$H_\mathrm{H} := \beta \sum_{k,q} \mathbf{S}_q \cdot \left(c_{k-q}^\dagger \boldsymbol{\sigma} c_k \right) \tag{3}$$

to localized magnetic moments $\mathbf{S}_r = \sum_q \mathbf{S}_q \, e^{\mathrm{i}qr}$ on sites r that are arranged in a one-dimensional chain. The conduction electrons induce an effective RKKY interaction between the localized spins

$$H_\mathrm{RKKY} := -\frac{2\beta^2}{A^2} \sum_q \sum_{a,b=1}^{3} S_{-q;a} \, \chi_{q;a,b} \, S_{q;b}, \tag{4}$$

where A is a constant with the dimension of area that characterizes the extend of the electron wave-function perpendicular to the chain and $\chi_{q;a,b}$, $a, b = 1, 2, 3$, is the static magnetic susceptibility tensor of the electrons.

We now assume a geometry in which the conduction electrons originate from a two-dimensional surface on which the magnetic spins are positioned, that has its normal in the 3-direction. The Rashba spin-orbit coupling due to the inversion symmetry breaking by the surface takes the form $\mathbf{g}_\mathbf{k} \propto \mathbf{e}_3 \wedge \mathbf{k}$, with $\mathbf{e}_3$ the unit vector in 3-direction. If we further assume that the chain of magnetic moments stretches along the 1-direction on the surface, so that $k \equiv k_1$, the spin-orbit coupling term in Hamiltonian (1) takes the from $\mathbf{g}_k = (0, \alpha \, k, 0)^\mathsf{T}$. The electron's spin susceptibility is then

$$\chi_{q;ab} \propto \int \mathrm{d}k \int \mathrm{d}\omega \mathrm{tr} \left[\sigma_a G_{k+q,\omega} \sigma_b G_{k,\omega} \right], \tag{5}$$

where $G_{k+q,\omega}$ is the Green function corresponding to Hamiltonian (1). Hamiltonian (1) has a spin rotation symmetry if $\mathbf{g}_\mathbf{k} \propto \mathbf{e}_3 \wedge \mathbf{k}$ for rotations around the 2-direction in spin space $\mathcal{R}_2(\varphi)$ by an

arbitrary angle $\varphi \in [0, 2\pi)$, i.e., $\mathcal{R}_2(\varphi) H_0 \mathcal{R}_2^{-1}(\varphi) = H_0$, and so has the Green function. As a consequence, for $b = 1, 3$,

$$\begin{aligned}
\chi_{q;2b} &\propto \int \mathrm{d}k \int \mathrm{d}\omega \mathrm{tr}\left[\sigma_a \mathcal{R}_2(\pi) G_{k+q,\omega} \mathcal{R}_2^{-1}(\pi) \sigma_b \mathcal{R}_2(\pi) G_{k,\omega} \mathcal{R}_2^{-1}(\pi)\right] \\
&= \int \mathrm{d}k \int \mathrm{d}\omega \mathrm{tr}\left[\sigma_2 G_{k+q,\omega} \mathcal{R}_2^{-1}(\pi) \sigma_b \mathcal{R}_2(\pi) G_{k,\omega}\right] \\
&= -\int \mathrm{d}k \int \mathrm{d}\omega \mathrm{tr}\left[\sigma_2 G_{k+q,\omega} \sigma_b G_{k,\omega}\right] \\
&\propto -\chi_{q;2b},
\end{aligned} \tag{6}$$

where we used $\mathcal{R}_2^{-1}(\pi) \sigma_b \mathcal{R}_2(\pi) = -\sigma_b$ for $b = 1, 3$. Thus, the components $\chi_{q;21}$, $\chi_{q;23}$, $\chi_{q;12}$, and $\chi_{q;32}$ vanish for reasons of symmetry. The remaining components of the electron's spin susceptibility can be computed from

$$\hat{\chi}_q = \frac{1}{N} \sum_k \sum_{\lambda,\lambda'=\pm} \begin{pmatrix} 1 - s_{k(k+q)}\lambda\lambda' & 0 & \mathrm{i}(\lambda s_k - \lambda' s_{k+q}) \\ 0 & 1 + s_{k(k+q)}\lambda\lambda' & 0 \\ -\mathrm{i}(\lambda s_k - \lambda' s_{k+q}) & 0 & 1 - s_{k(k+q)}\lambda\lambda' \end{pmatrix} \frac{f_{\mathrm{FD}}(\xi_{k,\lambda}) - f_{\mathrm{FD}}(\xi_{k+q,\lambda'})}{\xi_{k+q,\lambda'} - \xi_{k,\lambda}}, \tag{7}$$

where N is the number of lattice sites, f_{FD} is the Fermi-Dirac distribution, and $s_k := \operatorname{sign} k$. Two observations are in order. (i) In absence of Rashba spin-orbit interaction ($\xi_{k,\lambda} = \xi_{k,\lambda'}$), the susceptibility tensor takes the SU(2) invariant form $\hat{\chi}_q = \mathbb{1}\, \chi_q$. In this case χ_q features a divergence at $q = 2k_{\mathrm{F}} := 2\sqrt{2m\mu}$ which leads to a helical magnetic ordering with that wavevector. The magnetic order spontaneously breaks both the continuous SU(2) rotation symmetry in spin space as well as the discrete translational symmetry. (ii) With finite Rashba spin-orbit coupling, the diagonal entries of the 1 and 3 components remain equal (degenerate) and are coupled by off-diagonal matrix elements of definite helicity.

Numerical evaluation of the nonvanishing matrix elements of $\hat{\chi}_q$ shows that $\chi_{q;11} = \chi_{q;33}$ develops a double peak structure with one peak each at

$$q_\pm := 2\sqrt{2m\mu + m^2\alpha^2} \pm 2m\alpha, \tag{8}$$

while $\chi_{q;22}$ retains a single peak at $q_0 := 2\sqrt{2m\mu + m^2\alpha^2}$ [Consult Supplementary Figure 1 (c)-(e) for the physical significance of these momenta.]. The purely imaginary off-diagonal terms $\chi_{q;13} = -\chi_{q;31}$ are peaked at $q_\pm$ as well and change sign between the peaks $\chi_{q_+;13} = -\chi_{q_-;13}$ [see Supplementary Figure 1 (b)].

The dominant magnetic instability is determined by the peak structure of the magnetic susceptibility [see Supplementary Figure 1 (b)]. At first sight, it seems that the peak in $\chi_{q,22}$ is far exceeding all other peaks and one would suspect a spin-density wave order with q_0 would form, hybridizing the bands as shown in Supplementary Figure 1 (c). However, the susceptibilities for the helical orders in the 1-3-plane are in fact $\chi_{q,11} \pm \mathrm{i}\chi_{q,13}$ and we observe from Supplementary Figure 1 (b) that they have degenerate peak heights. [Precise definitions of these magnetic orders are given in the next section.]

B. The classical magnetic order

We now want to find the classical ground state of Hamiltonian (4). To be able to make analytical progress, we make simplifying assumptions about the form of the susceptibility tensor. For one, Eq. (6) enforces by symmetry zeros in the susceptibility tensor that render it block-diagonal, decoupling the 2-components of the magnetic moments from the 1- and 3-components of the magnetic moments, with the latter mutually coupled. We shall approximate the various peaks in the susceptibility by a delta function in momentum space.

We want to solve for the spin configuration that minimizes the energy of Hamiltonian (4) if the local constraint

$$\mathbf{S}_r^2 = 1, \quad \forall r = 1, \cdots, L, \tag{9}$$

is imposed on each site r of the chain of length L for the two cases

$$\begin{aligned} &\text{(A)} \qquad \chi_{q,22} = \frac{\bar{\chi}}{2}(\delta_{q-q_0} + \delta_{q+q_0}), \\ &\text{(B)} \qquad \chi_{q,11} = \chi_{q,33} = \frac{\bar{\chi}_{11}}{2}(\delta_{q-q_+} + \delta_{q+q_+}), \\ &\qquad\qquad \chi_{q,13} = \chi_{q,31}^{*} = -\frac{\bar{\chi}_{13}}{2}(\delta_{q-q_+} - \delta_{q+q_+}), \end{aligned} \tag{10}$$

with all components not listed vanishing in either case.

1. *Case (A): Antiferromagnet*

In case (A), in view of Hamiltonian (4), the task is to maximize the value of $|S_{2;q_0}|^2$ for spin configurations subject to the constraint (9). The Fourier component of a spin configuration $S_{2;r} = \pm 1$ for all sites r is computed as

$$S_{2;q_0} = \frac{1}{L}\sum_r e^{-\mathrm{i}q_0 r} S_{2;r}. \tag{11}$$

Consider without loss of generality the case where $S_{2;q_0} = S_{2;-q_0}$ is real,

$$\frac{S_{2;q_0} + S_{2;q_0}}{2} = \frac{1}{L}\sum_r \cos(q_0 r) S_{2;r}. \tag{12}$$

To maximize the left hand side, we have to choose

$$S_{2;r} = \mathrm{sign}[\cos(q_0 r)]. \tag{13}$$

This configuration has the Fourier component of size

$$S_{2;q_0} = \frac{2}{\pi} \approx 0.637 \tag{14}$$

and the energy is

$$E_{\mathrm{af}} = -\frac{2\beta^2}{A^2}\left(\frac{2}{\pi}\right)^2 \bar{\chi} \approx -\frac{2\beta^2}{A^2} 0.405\bar{\chi}. \tag{15}$$

Note that this is in particular smaller than the energy $E_{\mathrm{af}} \approx -\frac{2\beta^2}{A^2}0.25\bar{\chi}$ of a spin-density wave $S_{2;r} = \cos(q_0 r)$ [which does not satisfy the local constraint (9)].

2. *Case (B): Helix*

To analyze case (B), we have to face the problem that the local constraint (9) is not conveniently written in momentum space. To circumvent this, we replace it by the global constraint

$$\sum_r \mathbf{S}_r^2 = L \tag{16}$$

and check a posteriori that the solution we obtained also satisfies the local constraint. We thus consider the Hamiltonian

$$\begin{aligned} H_{\mathrm{helix}} :=& -\frac{2\beta^2}{A^2}\left[\bar{\chi}_{11} S_{-q_+;1} S_{q_+;1} + \bar{\chi}_{11} S_{-q_+;3} S_{q_+;3} - \bar{\chi}_{13}(S_{-q_+;1} S_{q_+;3} - S_{-q_+;3} S_{q_+;1})\right] \\ & - \lambda \sum_q \mathbf{S}_{-q}\cdot\mathbf{S}_q, \end{aligned} \tag{17}$$

where λ is a Lagrange multiplier.

Minimization yields

$$\begin{aligned} 0 &= \frac{\partial H_{\mathrm{helix}}}{\partial S_{\pm q_+;1}} = -\frac{2\beta^2}{A^2}(\bar{\chi}_{11} S_{\mp q_+;1} \pm \bar{\chi}_{13} S_{\mp q_+;3}) - \lambda S_{\mp q_+;1}, \\ 0 &= \frac{\partial H_{\mathrm{helix}}}{\partial S_{\pm q_+;3}} = -\frac{2\beta^2}{A^2}(\bar{\chi}_{11} S_{\mp q_+;3} \mp \bar{\chi}_{13} S_{\mp q_+;1}) - \lambda S_{\mp q_+;3}, \\ 0 &= \frac{\partial H_{\mathrm{helix}}}{\partial \lambda} = -\sum_q \mathbf{S}_{-q}\cdot\mathbf{S}_q. \end{aligned} \tag{18}$$

A solution to Eq. (18) is given by

$$S_{\pm q_+;1} = \frac{1}{2}, \qquad S_{\pm q_+;3} = \pm\frac{1}{2\mathrm{i}}, \qquad \lambda = -\frac{2\beta^2}{A^2}(\bar{\chi}_{11} + \mathrm{i}\bar{\chi}_{13}). \tag{19}$$

This is nothing but a helix in position space

$$\mathbf{S}_r = (\cos(q_+ r), 0, \sin(q_+ r))^{\mathsf{T}}, \tag{20}$$

which automatically satisfies the local constraint (9) as well. The energy of this minimizing solution is

$$E_{\mathrm{helix}} = -\frac{2\beta^2}{A^2}\frac{\bar{\chi}_{11} + \mathrm{i}\bar{\chi}_{13}}{2}, \tag{21}$$

which is lower than the energy (15), given the observation that $\bar{\chi}_{11} + \mathrm{i}\bar{\chi}_{13} = \bar{\chi}$.

The same arguments for the wave vector q_- yield the conclusion that a helix with opposite helicity

$$\mathbf{S}_r = (\cos(q_- r), 0, -\sin(q_- r))^{\mathsf{T}}, \tag{22}$$

is an energetically degenerate state.

We conclude that the magnetic state that minimizes the energy is given by one of the degenerate helices (20) and (22). The back-action of this helical magnetic order on the itinerant electronic states is the opening of a hybridization gap between two of the four electronic branches that cross the Fermi level [see Supplementary Figure 1 (d) and (e)]. We are thus left with a single species of effectively spineless electrons. When superconducting paring is induced on them by proximity, they are bound to form a topological superconductor (in this toy unrealistic model of 1D RKKY interaction). One might wonder how the presence of superconductivity alters the RKKY mechanism presented here. In fact, as long as the superconducting order parameter is smaller than the energy scale associated with the Rashba spin-orbit coupling ($\Delta \ll \alpha k_{\mathrm{F}}$), its effect is merely to round off the peaks in the magnetic susceptibility, leaving the qualitative results unaltered.

C. The effect of a finite in-plane magnetic field

In anticipation of the crucial role that an externally applied homogeneous magnetic field will play in our proposal, we want to study whether such a field will change the nature of the magnetic order. In particular, we are interested in applying a field in the 1-2 plane. This field does not destroy the superconductivity of a thin layer in the 1-2 plane, since the orbital repairing is not

effective. We use the parametrization

$$\mathbf{B} = B(\cos\varphi, \sin\varphi, 0), \tag{23}$$

which essentially alters the electronic structure via Zeeman coupling according to the substitution

$$\mathbf{g}_k = (B\cos\varphi, B\sin\varphi + \alpha k, 0) \tag{24}$$

in Hamiltonian (1). Our strategy is to compute the magnetic susceptibility in the presence of the magnetic field (23) and to deduce possible changes in the magnetic order from the modifications in the peak structure. Such changes may indicate transitions between phases of qualitatively different magnetic order that might be induced by the external magnetic field at some critical field strength B_c.

Studying the evolution of the Fermi points as a function of φ and B can already give a hint. At the magnetic field determined by the Rashba spin-orbit coupling energy scale

$$B_c = \sqrt{2m\mu}\alpha \tag{25}$$

two of the four Fermi points at positive (negative) momenta become degenerate for the field orientation $\varphi = \pi/2$ ($\varphi = 3\pi/2$).

A numerical evaluation of the magnetic susceptibility is shown in in Supplementary Figure 2. For simplicity, the susceptibility is only shown for the special field orientations $\varphi = 0$ and $\varphi = \pi/2$, but the following discussion is also compatible with the interpolation between these cases. For $B < B_c$, the changes in the magnetic susceptibility are small, suggesting that the helical order in the 1-3 plane is stable [see Supplementary Figure 2 (a)]. As B approaches B_c, at $\varphi = 0 \bmod \pi$ an extra peak appears in χ_{33} at q_0 [see panel (b) from Supplementary Figure 2]. This, together with the already existing peak of χ_{22} at q_0 poses the possibility of helical magnetic order in the 2-3 plane, that competes with the order in the 1-3 plane. At the same value of the magnetic field, but for $\varphi = \pi/2 \mathrm{mod}\pi$, the single peak in χ_{22} at $2k_F$ splits and might allow for helical order in the 1-2 or 2-3 plane. However, since the components χ_{12} and χ_{23} are much smaller than χ_{13} (and in fact very close to zero in Supplementary Figure 2), the helix in the 1-3 plane is likely to remain the dominant instability at this angle of the magnetic field.

When B exceeds B_c, at $\varphi = 0 \bmod \pi$ both χ_{22} and χ_{33} are dominated by a single peak at q_0 [see panel (c) from Supplementary Figure 2; q_0 is defined below Eq. (8)]. This, together with the absence of such a peak in χ_{11}, suggests that helical magnetic order in the 2-3 plane will form. At

the same value of the magnetic field, but for $\varphi = \pi/2 \text{mod} \pi$, a double-peak structure is eminent at the same wave vectors in χ_{11}, χ_{33}, and χ_{13}, while χ_{22} has a double-peak at different wave vectors, while the off-diagonal elements χ_{12} and χ_{23} nearly vanish. This suggests that a helical order in the 1-3 plane will persist at $\varphi = \pi/2$.

We can summarize these results as follows: A homogeneous magnetic field $\mathbf{B}$ will, in addition to inducing a homogeneous magnetization parallel to its direction, also modify the spatially varying (helical) component of the magnetic order. The critical scale that it has to exceed for qualitatively changing the helical order is given by B_{c} from Eq. (25). If $B < B_{\mathrm{c}}$, the helical component of the magnetization remains in the 1-3 plane, pinned by Rashba spin-orbit coupling and independent of the direction of $\mathbf{B}$. In contrast, if $B > B_{\mathrm{c}}$, the helical component of the magnetization will lie in the plane perpendicular to $\mathbf{B}$, i.e., in the 2-3 plane if $\mathbf{B}$ points in the 1-direction and in the 1-3 plane if $\mathbf{B}$ points in the 2-direction. This is summarized in the schematic phase diagram Supplementary Figure 3. For what follows, the important assumption that we derive from this phase diagram is that the helical magnetic order remains stable in the 1-3 plane for inplane magnetic fields $B < B_{\mathrm{c}}$ irrespective of their orientation φ. While the discussion above is largely speculative, the only thing we need for our setup is that the applied magnetic field influences the helix to a much less extent than it influences the induced p-wave gap on the chain. This is likely to be true, as the energy scale for the induced p-wave gap on the chain is much smaller than that of the magnetic atoms. [1]

Supplementary Note 2. Superconducting states in presence of helical magnetic order

A. Topological transitions in ferromagnetically ordered chains with Rashba spin-orbit coupling

We start by investigating the topological phase transition induced by an external magnetic field in a chain with a suitable combination of Rashba spin-orbit coupling and ferromagnetic order (or, alternatively, an external magnetic field as in the case of semiconductor nanowires). Let us consider the following Hamiltonian

$$\tilde{H} = \sum_{n=1}^{L} \left\{ c_n^{\dagger}(t + \mathrm{i}\alpha\,\sigma_2)c_{n+1} + \Delta c_{n,\uparrow}^{\dagger} c_{n,\downarrow}^{\dagger} + \text{h.c.} \right\} + \sum_{n=1}^{L} c_n^{\dagger} \left(\mathbf{B}\cdot\boldsymbol{\sigma} - \mu \right) c_n. \qquad (26)$$

Formally this Hamiltonian is different from Hamiltonian (34) only in that the magnetic ingredient is constant in space for the former, but position-dependent for the latter.

Hamiltonian (26) is gapless for $|B_2| > |\Delta|$. The gap that closes at $|B_2| = |\Delta|$ is an indirect gap for generic parameters (see Supplementary Figure 4). If gapped, the ground state of Hamiltonian (26) is topological if

$$\min(A_-, A_+) < |\mathbf{B}| < \max(A_-, A_+), \tag{27}$$

where $A_\pm := \sqrt{(2t \pm \mu)^2 + \Delta^2} > |\Delta|$.

We want to study topological phase transitions of Hamiltonian (26) as a function of $\mathbf{B}$ between gapped phases. For that reason, we will always require $|B_2| < |\Delta|$. As long as this condition is met, owing to particle-hole symmetry, a topological phase transition can only happen when a direct gap closes and reopens at the momenta $k = 0$ or π. These momenta are also inversion symmetric momenta at which the spin-orbit coupling term is vanishing. Therefore the Bloch Hamiltonian in the Nambu basis has the particularly simple form

$$\tilde{\mathcal{H}}(k = 0, \pi) = [(2t\cos k - \mu)\tau_3 + \Delta\tau_1] \otimes \sigma_0 + \tau_0 \otimes (\mathbf{B} \cdot \boldsymbol{\sigma}), \tag{28}$$

where τ's stand for the Pauli matrices for the particle-hole components. The spectrum of this Hamiltonian at $k = 0, \pi$ only depends on $|\mathbf{B}|$ instead of the orientation of $\mathbf{B}$, because for $\mathbf{B} = 0$ the Hamiltonian (28) has the full $SU(2)$ spin-rotation symmetry.

The eigenvalues of (28) are given by

$$\xi(k = 0) = \pm|\mathbf{B}| \pm A_-, \qquad \xi(k = \pi) = \pm|\mathbf{B}| \pm A_+, \tag{29}$$

where the signs are uncorrelated. Assuming a weak magnetic field, the direct gap closes when $|\mathbf{B}| = \min(A_-, A_+)$, irrespective of the orientation of the field. This immediately rules out the possibility of a topological phase transition induced by varying the orientation of a magnetic field while keeping its magnitude constant. However, this can still be achieved by applying a constant background magnetic field on top of the rotating one. For example, if $\mathbf{B} = (B_1 + B_2\cos\varphi, B_2\sin\varphi, 0)$, with $|B_1| + |B_2| > \min(A_-, A_+) > \sqrt{B_1^2 + B_2^2}$, two topological phase transitions will occur when φ is tuned from 0 to 2π (see Supplementary Figure 5).

B. Superconducting phase of a straight chain with helical magnetic order

We are now going to investigate the one-dimensional electronic model of the chain subject to proximity-induced superconducting pairing, the helical magnetic order and Rashba spin-orbit

coupling as well as an in-plane Zeeman field. We assume that the classical helical magnetic order discussed in the last section is rigidly formed and couples via a Hunds-type coupling to the conduction electrons. Given the helical magnetic order, the Rashba spin-orbit coupling is not a physically necessary ingredient for the effects that we are interested in, but we still include it for a more complete tight-binding Hamiltonian. Consider the following model Hamiltonian for a straight chain of L atoms

$$H = \sum_{n=1}^{L} \left\{ c_n^\dagger (t + \mathrm{i}\alpha\,\sigma_2) c_{n+1} + \Delta c_{n,\uparrow}^\dagger c_{n,\downarrow}^\dagger + \text{h.c.} \right\} + \sum_{n=1}^{L} c_n^\dagger \left[(\mathbf{B} + \mathbf{M}_n) \cdot \boldsymbol{\sigma} - \mu \right] c_n, \tag{30}$$

where $c_n^\dagger = (c_{n,\uparrow}^\dagger, c_{n,\downarrow}^\dagger)$ and $c_{n,s}^\dagger$ creates an electron of spin $s = \uparrow, \downarrow$ on site $n = 1, \cdots, L$. Here, t is the nearest-neighbor hopping integral, α parametrizes the Rashba spin-orbit coupling, Δ is the superconducting gap, μ is the chemical potential, and $\mathbf{B}$ is the external magnetic field. As discussed in the last section, the magnetic moment of the helical order lies in the 1-3 plane and has the spatial dependence

$$\mathbf{M}_n = M[\cos(n\,\theta + \theta_0), 0, \pm\sin(n\,\theta + \theta_0)]^\mathsf{T}, \tag{31}$$

where M is the overall amplitude, θ is the tilt between adjacent moments, $\pm$ stands for the two possible helicities and θ_0 is a phase shift. If the pitch θ between adjacent moments is large (such as $\pi/3$ or $\pi/4$), the choice of phase θ_0 can have profound consequences on the spectrum of Hamiltonian (30) (see Supplementary Figure 6). For small θ, in contrast, the choice of θ_0 is inconsequential as the system can be (approximately, up to corrections of order $\theta_0/2\pi$) transformed to $\theta_0 = 0$ by an appropriately chosen translation.

Before we proceed, we shall briefly illustrate that the helical magnetic order is gauge equivalent to the Rashba spin-orbit coupling plus a homogeneous Zeeman field in 3-direction. To see that, we perform a n-dependent unitary rotation U_n on $c_n = U_n \tilde{c}_n$ which is defined by

$$U_n^\dagger (\mathbf{M}_n \cdot \boldsymbol{\sigma}) U_n = M\,\sigma_3 \tag{32}$$

and represented as

$$U_n = \exp(-\mathrm{i}n\,\theta\,\sigma_2/2). \tag{33}$$

If the Zeeman field $\mathbf{B} = (0, B_2, 0)$ points in the 2-direction, the Hamiltonian reads in terms of the

transformed fermion operators

$$H = \sum_{n=1}^{L} \left\{ \tilde{c}_n^\dagger (\tilde{t} + \mathrm{i}\tilde{\alpha}\,\sigma_2)\tilde{c}_{n+1} + \Delta \tilde{c}_{n,\uparrow}^\dagger \tilde{c}_{n,\downarrow}^\dagger + \text{h.c.} \right\} + \sum_{n=1}^{L} \tilde{c}_n^\dagger \left(B_2\,\sigma_2 + M\,\sigma_3 - \mu \right) \tilde{c}_n, \tag{34}$$

with

$$\tilde{t} := t\,\cos\frac{\theta}{2} + \alpha\,\sin\frac{\theta}{2}, \qquad \tilde{\alpha} := \alpha\,\cos\frac{\theta}{2} - t\,\sin\frac{\theta}{2}. \tag{35}$$

All spatial dependence of the matrix elements has been gauged away and traded for a renormalized hopping and Rashba spin-orbit coupling. Depending on the combination of the parameters, the gap opened by the superconducting order parameter can either be topological or trivial, in the sense that each end of the chain hosts a single Majorana bound state at zero energy or not.

1. *Topological and trivial phase of the model*

Let us examine possible phase transitions for the model in absence of a magnetic field $\mathbf{B} = 0$ using the representation (34). The fact that the helical magnetic order is a Fermi surface instability (say $\theta = q_\lambda$, $\lambda = \pm$) in absence of superconducting pairing produces the following implicit equation for θ

$$0 = 2t\,\cos\frac{\theta}{2} + \lambda 2\alpha\,\sin\frac{\theta}{2} - \mu. \tag{36}$$

Equation (36) is the eigenvalue equation $\xi_{\theta/2} = 0$ of Hamiltonian (30) for $\Delta = \mathbf{B} = \mathbf{M}_n = 0$, where ξ_k is the eigenvalue at momentum k and the condition $\xi_k = 0$ determines the Fermi momenta. It is the lattice analogue of Eq. (8). Here, we considered the unrealistic but analytically tractable situation of the 1D RKKY interaction as the origin of the helix. We note that in the realistic 2D RKKY case [2], where the helix configuration depends on the Rashba spin-orbit coupling, tantamount but similar physics takes place. The dispersion of Hamiltonian (34) reads for $B_2 = 0$

$$\tilde{\xi}_k = \pm\sqrt{M^2 + \Delta^2 + C_k^2 + S_k^2 \pm 2\sqrt{C_k^2 S_k^2 + M^2\left(\Delta^2 + C_k^2\right)}}, \tag{37}$$

where $C_k \equiv 2\tilde{t}\cos\,k - \mu$ and $S_k \equiv 2\tilde{\alpha}\sin\,k$. At $k = 0$, the effective Hamiltonian takes a Dirac form for two dispersing modes with masses

$$\tilde{\xi}_0 = \pm\,M \pm \sqrt{\Delta^2 + (2\tilde{t} - \mu)^2}. \tag{38}$$

There is a topological phase transition when either of these masses changes sign, namely at $|M| = \sqrt{\Delta^2 + (2\tilde{t} - \mu)^2}$.

In contrast, at $k = \pi$, the energies are

$$\tilde{\xi}_\pi = \pm M \pm \sqrt{\Delta^2 + (2\tilde{t} + \mu)^2}, \tag{39}$$

and hence there is a phase transition at $|M| = \sqrt{\Delta^2 + (2\tilde{t} + \mu)^2}$. Assuming without loss of generality that $|2\tilde{t} - \mu| < |2\tilde{t} + \mu|$, this yields the following condition for being in the *topological phase*:

$$\sqrt{\Delta^2 + (2\tilde{t} - \mu)^2} < M < \sqrt{\Delta^2 + (2\tilde{t} + \mu)^2}. \tag{40}$$

2. *Topological phase transition with* B_2

As the Rashba spin-orbit coupling, the magnetic field B_2 enters the Hamiltonian by multiplying the second Pauli matrix. In effect, B_2 thus shifts $\sin k \to -B_2/\alpha + \sin k$, removing the spectral degeneracy between k and $-k$. Since the formation of Cooper pairs is energetically disfavored if the participating electrons are not at momenta k and $-k$, B_2 will generically drive a phase transition into a gapless state (with indirect band gap), iff

$$|B_2| > |\Delta| \qquad \text{gapless} \tag{41}$$

independent of M, as long as $|M| < |2\tilde{t} + \mu|$. Here, we assume $|2\tilde{t} - \mu| < |2\tilde{t} + \mu|$ and $(2\tilde{t} - \mu)(2\tilde{t} + \mu) > 0$.

To see how Eq. (41) comes about, we note that the condition for a zero-energy eigenvalue of Hamiltonian (34) is given by

$$D(k) = (S_k^2 + C_k^2 - M^2 + \Delta^2 - B_2^2)^2 - 4S_k^2(C_k^2 - M^2) = 0, \tag{42}$$

where $C_k \equiv 2\tilde{t}\cos k - \mu$ and $S_k \equiv 2\tilde{\alpha}\sin k$ are the same as in Eq. (37). The existence of a solution to this equation requires

$$S_k = 0,\ C_k^2 - M^2 + \Delta^2 - B_2^2 = 0, \tag{43}$$

$$\text{or} \quad S_k \neq 0,\ C_k^2 - M^2 \geq 0,\ \left(S_k \pm \sqrt{C_k^2 - M^2}\right)^2 = B_2^2 - \Delta^2. \tag{44}$$

Let us first focus on the latter case, where $k \neq 0$ or π, and the solutions always come in pairs as $\pm k$. Clearly a solution in this case can exist only if $B_2^2 - \Delta^2 \geq 0$. When $B_2^2 - \Delta^2 = 0$, as long as $|M| < |2\tilde{t}+\mu|$, we find that $|S_k| - \sqrt{C_k^2 - M^2} < 0$ when k approaches π, and $|S_k| - \sqrt{C_k^2 - M^2} > 0$ when k approaches the point of k where $C_k^2 - M^2 = 0$, which implies that at least one pair of

solutions of the original equation exist. By the same token, when $(2\tilde{t}+\mu)^2 - M^2 > B_2^2 - \Delta^2 > 0$, solutions of $k \neq 0$ or π still exist to the original equation (42). This proves the statement of Eq. (41).

In the case of Eq. (43), $k = 0$ or π, and the condition $|B_2| > |\Delta|$ is not necessary. Instead the existence of a zero-energy solution requires $\tilde{\xi}_0 = 0$ or $\tilde{\xi}_\pi = 0$ with

$$\begin{aligned} \tilde{\xi}_0 &= \pm \left[\sqrt{B_2^2 + M^2} - \sqrt{\Delta^2 + (2\tilde{t} - \mu)^2} \right], \\ \tilde{\xi}_\pi &= \pm \left[\sqrt{B_2^2 + M^2} - \sqrt{\Delta^2 + (2\tilde{t} + \mu)^2} \right]. \end{aligned} \tag{45}$$

That is, a direct gap closing is induced by the field B_2 at $k = 0$ or π, if

$$B_2^2 + M^2 = \Delta^2 + (2\tilde{t} \mp \mu)^2. \tag{46}$$

The regime that we are interested in is $|M| > |\Delta|, |B_2|$. In view of Eq. (40), no gap closing phase transition out of the topological phase can be induced that corresponds to $\tilde{\xi}_0 = 0$ by ramping up B_2. However, for suitable choice of chemical potential μ, B_2 can induce a topological phase transition at $\tilde{\xi}_\pi = 0$, while maintaining the condition $|B_2| < |\Delta|$ to escape entering the gapless phase. To sum up, for the parameters relevant to our proposal, we start from a topological phase by satisfying condition (40) and ramp up B_2 until one of the two things happen: Either, if $|M| > |2\tilde{t} + \mu|$, we enter a gapped topologically trivial phase at $B_2 = \sqrt{\Delta^2 + (2\tilde{t} + \mu)^2 - M^2}$. Or, if $|M| < |2\tilde{t} + \mu|$, we enter a gapless phase as soon as $|B_2| \geq |\Delta|$.

3. *Topological phase transition with* B_1

To study the effect of a homogeneous field B_1 in the 1-direction, the gauge transformation (33) is only partially useful, for it does not generate a Hamiltonian with the translational symmetry of the lattice. However, we can use it to move the position dependence from a large term in the Hamiltonian (i.e., M) to a small one (i.e., B_1). The transformed Hamiltonian reads

$$\tilde{H} = \sum_{n=1}^{L} \left\{ \tilde{c}_n^\dagger (\tilde{t} + \mathrm{i}\tilde{\alpha}\,\sigma_2) \tilde{c}_{n+1} + \Delta \tilde{c}_{n,\uparrow}^\dagger \tilde{c}_{n,\downarrow}^\dagger + \text{h.c.} \right\} + \sum_{n=1}^{L} \tilde{c}_n^\dagger \left(\tilde{\mathbf{B}}_n \cdot \boldsymbol{\sigma} + M\,\sigma_3 - \mu \right) \tilde{c}_n, \tag{47}$$

where the definitions of $\tilde{t}$ and $\tilde{\alpha}$ carry over from Eq. (35) and

$$\tilde{\mathbf{B}}_n = B_1 [\cos(n\,\theta + \theta_0), 0, \pm \sin(n\,\theta + \theta_0)]^\mathsf{T}, \tag{48}$$

with $\theta_0 = \pi/2$.

We can study it in the limit where both B_1 and Δ are small compared to the other energy scales. We will find that B_1 and Δ parametrize competing mass gaps of Hamiltonian (34). If Δ (B_1) dominates, the system is in the topological (trivial) phase. In absence of B_1, the Bloch Hamiltonian that corresponds to Eq. (34) in the basis $(c_{k,\uparrow}, c_{k,\downarrow}, c^\dagger_{-k,\uparrow}, c^\dagger_{-k,\downarrow})$ is given by

$$\mathcal{H}(k) = \begin{pmatrix} 2\tilde{t}\cos k - \mu + M & -2\mathrm{i}\tilde{\alpha}\sin k & 0 & \Delta \\ 2\mathrm{i}\tilde{\alpha}\sin k & 2\tilde{t}\cos k - \mu - M & -\Delta & 0 \\ 0 & -\Delta & -2\tilde{t}\cos k + \mu - M & 2\mathrm{i}\tilde{\alpha}\sin k \\ \Delta & 0 & -2\mathrm{i}\tilde{\alpha}\sin k & -2\tilde{t}\cos k + \mu + M \end{pmatrix}. \tag{49}$$

Linearized around the Fermi momentum k_{F} in absence of the superconducting gap (but with nonzero M), the Bloch Hamiltonian takes the general form

$$\mathcal{H}_{\mathrm{lin},\Delta}(p) = \begin{pmatrix} F + \frac{\tilde{t}}{\tilde{\alpha}}\sqrt{FG}\,p & \mathrm{i}\sqrt{FG} + \mathrm{i}Lp & 0 & \Delta \\ -\mathrm{i}\sqrt{FG} - \mathrm{i}Lp & G + \frac{\tilde{t}}{\tilde{\alpha}}\sqrt{FG}\,p & -\Delta & 0 \\ 0 & -\Delta & -F - \frac{\tilde{t}}{\tilde{\alpha}}\sqrt{FG}\,p & -\mathrm{i}\sqrt{FG} - \mathrm{i}Lp \\ \Delta & 0 & \mathrm{i}\sqrt{FG} + \mathrm{i}Lp & -G - \frac{\tilde{t}}{\tilde{\alpha}}\sqrt{FG}\,p \end{pmatrix}. \tag{50}$$

Here, $k = k_{\mathrm{F}} + p$ and F, G, and L are constants that depend on the parameters in Eq. (49). To linear order in Δ, the gap Δ_{lin} of $\mathcal{H}_{\mathrm{lin}}(p)$ at $p = 0$ is given by

$$\begin{aligned} \Delta_{\mathrm{lin}} &= \Delta\sqrt{\left|1 - \frac{(F-G)^2}{(F+G)^2}\right|} \\ &= \Delta\sqrt{\left|1 - \frac{4M^2(\tilde{t}^2 + \tilde{\alpha}^2)^2}{\left[\tilde{\alpha}^2\mu + \tilde{t}\,\sqrt{(M^2 + 4\tilde{\alpha}^2)(\tilde{t}^2 + \tilde{\alpha}^2) - \tilde{\alpha}^2\mu^2}\right]^2}\right|}. \end{aligned} \tag{51}$$

This has to be contrasted with the gap that is introduced by B_1. In this case, the effective Hamiltonian reads in the basis $(c_{k_{\mathrm{F}}+p,\uparrow}, c_{k_{\mathrm{F}}+p,\downarrow}, c_{k_{\mathrm{F}}+\theta+p,\uparrow}, c_{k_{\mathrm{F}}+\theta+p,\downarrow})$

$$\mathcal{H}_{\mathrm{lin},B_1}(p) = \begin{pmatrix} F + \frac{\tilde{t}}{\tilde{\alpha}}\sqrt{FG}\,p & \mathrm{i}\sqrt{FG} + \mathrm{i}Lp & \mathrm{i}B_1/2 & B_1/2 \\ -\mathrm{i}\sqrt{FG} - \mathrm{i}Lp & G + \frac{\tilde{t}}{\tilde{\alpha}}\sqrt{FG}\,p & B_1/2 & -\mathrm{i}B_1/2 \\ -\mathrm{i}B_1/2 & B_1/2 & F - \frac{\tilde{t}}{\tilde{\alpha}}\sqrt{FG}\,p & \mathrm{i}\sqrt{FG} - \mathrm{i}Lp \\ B_1/2 & \mathrm{i}B_1/2 & -\mathrm{i}\sqrt{FG} + \mathrm{i}Lp & G - \frac{\tilde{t}}{\tilde{\alpha}}\sqrt{FG}\,p \end{pmatrix}. \tag{52}$$

To linear order in B_1, the gap that emerges at $p = 0$ is given by

$$\begin{aligned}\Delta_{B_1} =& \frac{B_1}{2}\left|\frac{F-G}{F+G}\right| \\ =& \frac{B_1}{2}\left|\frac{M(\tilde{t}^2+\tilde{\alpha}^2)}{\tilde{\alpha}^2\mu + \tilde{t}\,\sqrt{(M^2+4\tilde{\alpha}^2)(\tilde{t}^2+\tilde{\alpha}^2)-\tilde{\alpha}^2\mu^2}}\right| .\end{aligned} \tag{53}$$

As Δ_{B_1} and Δ_{lin} are acting on different degrees of freedom (electrons with momenta differing by θ in one case and the electron-hole space in the other case), they are commuting and hence competing mass terms in an effective Dirac equation. Hence, the larger of Δ_{B_1} and Δ_{lin} determines the phase of the system. If $\Delta_{\text{lin}} > \Delta_{B_1}$, the superconductor is topological, hosting Majorana end states. If $\Delta_{\text{lin}} < \Delta_{B_1}$ it is trivial. Importantly, if parameters are such that

$$\left|\frac{F-G}{F+G}\right| \sim 1, \tag{54}$$

the gap Δ_{B_1} scales faster with B_1 than the gap Δ_{lin} scales with Δ. As a consequence, for some given superconducting gap Δ, we can expect a phase transition from the topological to the trivial superconductor at a critical field $B_{1,\text{c}}$ which is

$$B_{1,\text{c}} < \Delta. \tag{55}$$

4. *Phase diagram*

We have now sown that both B_1 and B_2 can induce a topological phase transition in the chain, based on very different mechanisms. Crucially, the critical field strength $B_{1,\text{c}}$ at which this phase transition appears is different from the critical field strength $B_{2,\text{c}}$. For appropriate choice of the parameters, the system enters a gapless phase at $B_2 = B_{2,\text{c}} = \Delta$, while a transition from the topological to the gapless phase triggered by B_1 can happen at $B_1 = B_{1,\text{c}} < \Delta$. This has the following consequence: If the field strength of the external field B is chosen such that $B_{1,\text{c}} < B < B_{2,\text{c}}$, then by rotating the external field in the 1-2 plane

$$\mathbf{B} = B(\cos\varphi, \sin\varphi, 0)^{\mathsf{T}}, \tag{56}$$

the chain has to undergo a topological-to-trivial phase transition for some $\varphi = \varphi_0$ between 0 and $\pi/2$ (see Figure 2 in the main text). At this transition the bulk of the chain becomes gapless and two Majorana states emerge. This phase transition occurs while the field remains in the 1-2 plane. It is therefore not harmful to the superconducting substrate if this is a thin film.

C. Spectral properties of a circle and finite size scaling

We now return to consider the chain with helical magnetic order. In order to move Majorana bound states in a controlled way, one would like to pin them to some mobile domain wall that marks the phase transition between a trivial and a topological state. The external field is fixed in angle and magnitude. However, one can utilize the directional dependence and obtain a domain wall between two chain segments that are at an angle different from π to one another. Depending on that angle and the field orientation, one segment could be in the topological, the other in the trivial phase. In this case the two host a Majorana state between them. More generally, one can join up many such segments into a bent chain or a circle. We assume the rotation plane of the helix is locally unaffected by the bending. That is, the magnetic moments locally lie in the plane that is spanned by the normal of the superconductor and the tangent to the bent chain. For the latter, the angle φ that enters the Hamiltonian (30) via the parametrization (56) becomes position dependent as $\varphi \to \varphi_n = 2\pi n/L$ and the boundary conditions are changed from open to periodic. To retain the same helical magnetic order in the circle as in the straight chain, the limit in which the radius of the circle is much larger than the wave length of the magnetic helix, i.e., $2\pi/L \ll \theta$, has to be assumed.

As shown schematically in Supplementary Figure 7, the circle is then capable of hosting 4 Majorana bound states. These bound states can be moved by changing the magnetic field orientation. One potential issue of this setup regards other possible bound states of the domain walls at nonzero energy, besides the topological Majorana bound states. Such excited domain wall states can be close in energy to the Majorana bound states in a large circle, and will be the major threat to an adiabatic operation on the Majorana qubits. Therefore it is crucial to compare the scaling behavior of the Majorana splitting energy (E_M) and that of the first (domain-wall) excitation energy ($E^{(1)}_{\text{ex.}}$). Straightforward numerical diagonalization of the problem shows that E_M decays exponentially with an increasing circumference L [see Supplementary Figure 8(a)], whereas $E^{(1)}_{\text{ex.}}$ changes in a much slower rate as $1/\sqrt{L}$ [see Supplementary Figure 8(b)]. This drastic difference is due to the different origin of these two energies: E_M is a result of a finite overlap between the exponential tails of different Majorana wave-functions, while $E^{(1)}_{\text{ex.}}$ is related to the *local* spatial profile of the domain wall. In the next subsection we will analyze these behaviors in more detail by using an effective model for the domain wall. It is important to notice that in a circle of several hundreds of sites, $E_M \sim 10^{-5}\Delta$ while $E^{(1)}_{\text{ex.}} > 10^{-2}\Delta$, which leaves enough room for time/energy scales of

quasi-adiabatic processes in between.

D. Domain wall states in a circle

In this section, we would like to study the low-energy excitations above the Majorana states in an effective model that can be treated analytically. We anticipate that these lowest excitations at finite energy are localized at the same position as the Majorana bound states. It is convenient to introduce a continuous coordinate

$$x := \frac{\varphi - \varphi_0}{2\pi} L, \tag{57}$$

where L is the circumference of the circle and φ is angular coordinate measured relative to the orientation of the external $\mathbf{B}$ field. As discussed before, the Majorana state is localized at the critical angle φ_0, which corresponds to $x = 0$. We work in the continuum limit in which we can formulate an effective theory in the coordinate x instead of the lattice site labels n, provided that L is much larger than all other length scales (in particular the wavelength of the helical magnetic order) of the chain. In this limit, the generic low-energy effective Hamiltonian for excitations near $x = 0$ reads (see Supplementary Figure 9)

$$H = \begin{pmatrix} -\hbar v(i\partial_x + k_0) & \Delta'(x) \\ \Delta'(x) & \hbar v(i\partial_x + k_0) \end{pmatrix}. \tag{58}$$

We assume around the domain wall, the low-energy physics is dominated by states of momenta close to k_0, namely the momentum where the bulk gap closes and reopens at field angle φ_0 in the straight chain. We linearize the dispersion relation at the domain wall center, with respect to $k = k_0$, with a group velocity v. Finally, in writing Hamiltonian (58), we assumed that the gap parameter Δ' around the domain wall changes linearly with respect to the angle φ between the tangent line of the circle and the magnetic filed direction [cf. Supplementary Figure 10(a)]. Translated to the local coordinate we have chosen, this means $\Delta'(x) = sx/r$, where $s = \partial\Delta'/\partial\varphi$ and $r = L/2\pi$ is the radius of the circle.

Let us set $\hbar = 1$, and choose $k_0 = 0$ for the moment. The above Hamiltonian can be rewritten and transformed into a more convenient form

$$H = \sqrt{sv/r} \begin{pmatrix} & \tilde{x} - \partial_{\tilde{x}} \\ \tilde{x} + \partial_{\tilde{x}} & \end{pmatrix}, \quad \tilde{x} \equiv \sqrt{\frac{s}{vr}} x. \tag{59}$$

The solutions to this Hamiltonian are well-known in the context of graphene. The same Hamiltonian applies to the Landau level problem of a single species of Dirac electrons subject to an out-of-plane magnetic field. By this analogy, s/r is equivalent to the strength of the magnetic field.

The Hamiltonian (59) has one zero energy eigenstate, the Majorana bound state, given by

$$\Psi_0(\tilde{x}) \propto \exp(-\tilde{x}^2/2) \begin{pmatrix} 1 \\ 0 \end{pmatrix} . \tag{60}$$

In the original coordinate, this solution becomes

$$\Psi_0(x) = \left(\frac{s}{\pi v r}\right)^{1/4} \exp(-\frac{s}{2vr}x^2) \begin{pmatrix} 1 \\ 0 \end{pmatrix} , \tag{61}$$

where the wavefunction has been normalized in $(-\infty, +\infty)$.

The Majorana coupling energy E_M is then given proportional to

$$E_M \propto \sqrt{\frac{s}{\pi v r}} \int_{-\infty}^{+\infty} e^{-\frac{s}{2vr}[x^2+(x-\varphi_0 r)^2]} dx = e^{-\frac{s}{4v}\varphi_0^2 r}, \tag{62}$$

where φ_0 is the critical angle for the topological phase transition. Here, we assume without loss of generality $\varphi_0 < \pi/4$. Equation (62) shows that E_M indeed decays exponentially with an increasing size of the circle. Moreover, it provides a prediction for the decay length, which is examined by numerics as follows. First we extract s and φ_0 by using a linear fit to bulk band spectra as shown in Supplementary Figure 10(a), where we have fixed all parameters but the magnitude of the external magnetic field B. Next we extract the corresponding inverse decay length a from Supplementary Figure 8(a). Then we plot the correlation between a and $s\varphi_0^2$ in Supplementary Figure 10(b). Clearly, there is a remarkable agreement between the numerical results and the prediction of the effective model.

Another important length scale that can be derived from the effective model is the oscillation period of the Majorana wave-functions. This simply follows from the fact that the solutions of the Schrödinger equation when $k_0 \neq 0$ can be obtained from those when $k_0 = 0$ by substitution $\Psi \to \Psi \exp(ik_0 x)$. In the system we are considering, particle-hole symmetry dictates k_0 to be 0 or the Brillouin zone boundary $\pm\theta/2$, because otherwise k_0 must appear in pairs and the system will not undergo a topological phase transition; the specific mechanism for the phase transition that we have discussed further limits k_0 down to $\pm\theta/2$. In this case the solutions with factors $\exp(\pm i\theta x/2)$

must be superimposed to yield $\sin(\theta x/2)$ or $\cos(\theta x/2)$. Therefore the oscillation period for the wave function amplitude is $4\pi/\theta$, while the period of the probability is $2\pi/\theta$.

In fact, all the excited-state solutions to Hamiltonian (59) can be readily obtained. Of particular importance to us, concerning the adiabaticity of the manipulations on the Majorana states, is the first excited state

$$E^{(1)}_{\text{ex.}} = \sqrt{2sv/r}, \quad \Psi^{(1)}_{\text{ex.}}(\tilde{x}) \propto \exp(-\tilde{x}^2/2) \begin{pmatrix} \sqrt{2}\tilde{x} \\ 1 \end{pmatrix}. \tag{63}$$

Evidently, its energy scales as $1/\sqrt{r}$, which is analogous to the $\sqrt{B}$ dependence of the Landau levels in graphene. This energy scaling behavior is a local property of the domain wall, in contrast to the nonlocal nature of the Majorana coupling energy, and will dominate as long as the domain walls are sufficiently separated. We examine this power-law scaling behavior in comparison with numerical results, shown in Supplementary Figure 8(b), and again find a good agreement.

Supplementary Note 3. Braiding of Majorana states with the magnetic field

A set of N localized, well separated Majorana states with second-quantized operators $\gamma_i = \gamma_i^\dagger$, $i = 1, \cdots N$ that obey $\{\gamma_i, \gamma_j\} = 2\delta_{ij}$ furnish, asymptotically for large N, a $\sqrt{2}^N$-dimensional fermionic Hilbert space. Fermionic operators are pairwise linear combinations $2c_{ij} := \gamma_i + \mathrm{i}\gamma_j$, $2c_{ij}^\dagger = \gamma_i - \mathrm{i}\gamma_j$, of these Majorana operators. For example, two Majorana states γ_1 and γ_2, that are energetically separated from other excited states of the system, form a two-level system with eigenstates $|0\rangle$ and $|1\rangle = c_{12}^\dagger|0\rangle$, where the vacuum obeys $c_{12}|0\rangle = 0$. The states $|0\rangle$ and $|1\rangle$ have Fermion parity $P_{12} := (-1)^{c_{12}^\dagger c_{12}} = -\mathrm{i}\gamma_1\gamma_2$ of 1 and -1, respectively. An operation that changes the Fermion parity would thus change the state of this two-level system (qubit). However, parity-changing operations cannot be carried out by topological (braiding) operations. Rather, a topological qubit should be defined on a sector of constant parity. To realize this, one needs four Majorana modes to constitute a qubit. Defining the parity eigenstates of the first and second pair or Majorana modes as $|0\rangle_{12}$, $|1\rangle_{12}$ and $|0\rangle_{34}$, $|1\rangle_{34}$, respectively, we can define two states

$$|\bar{0}\rangle = |0\rangle_{12} \otimes |0\rangle_{34}, \qquad |\bar{1}\rangle = |1\rangle_{12} \otimes |1\rangle_{34}, \tag{64}$$

as a two level system. Both of these states have the same total parity $P_{12}P_{34} = +1$. States of odd parity are in principle degenerate, but cannot be accessed by the (topological) braiding operations.

We will now show how the elementary gate operations, namely the braid

$$|\bar{0}\rangle \to |\bar{0}\rangle, \qquad |\bar{1}\rangle \to -|\bar{1}\rangle, \tag{65}$$

and the σ_x gate

$$|\bar{0}\rangle \to |\bar{1}\rangle, \qquad |\bar{1}\rangle \to |\bar{0}\rangle. \tag{66}$$

can be implemented using the helical magnetic chain by an operation as simple as rotating the external magnetic field.

A. Two ellipses – the σ_x gate

To implement a σ_x gate with braiding operations, a four-Majorana qubit defined in Eq. (64) has to be used. It is implemented by braiding particle 1 around particle 3 (or equivalent processes). Interestingly, this can be achieved by using two overlapping ellipses with appropriately chosen critical angles φ_0 via a 2π rotation of the external magnetic field (see Supplementary Figure 11 and the corresponding Figure in the main text). In this geometry, there are eight Majorana states present.

For a given arrangement of ellipses, we can find out which Majorana state braids around which other one by analyzing diagrams such as Supplementary Figure 11(b), (d), and (f). We will explain their meaning in the following. Consider two ellipses with equal sizes of the principal axes p and q that are at an angle of $\pi/2$ to each other. The two ellipses are defined by the two conditions

$$1 = \frac{x_1^2}{p^2} + \frac{x_2^2}{q^2}, \qquad 1 = \frac{(x_1 - y_1)^2}{q^2} + \frac{(x_2 - y_2)^2}{p^2}, \tag{67}$$

where $\mathbf{y} = (y_1, y_2)$ is their relative displacement. Let us define a pair of angles $\tan\theta := px_2/(qx_1)$ and $\tan\gamma := q(x_2 - y_2)/[p(x_1 - y_1)]$ for the ellipses centered at 0 and $\mathbf{y}$, respectively. The angle φ of the tangent of the the first ellipse at θ with respect to the x_1-axis is determined by

$$\begin{aligned}\tan\left(\varphi + \frac{\pi}{2}\right) =& \frac{\mathrm{d}x_2}{\mathrm{d}x_1} \\ =& -\frac{q}{p}\frac{1}{\sqrt{1 - x_1^2/p^2}}\frac{x_1}{p} \\ =& -\frac{q^2}{p^2}\frac{x_1}{x_2} \\ =& -\frac{q}{p}\frac{1}{\tan\theta},\end{aligned} \tag{68}$$

and analogously for the tangent to the second ellipse at γ . Therefore,

$$\tan\theta = \frac{q}{p}\tan\varphi, \qquad \tan\gamma = \frac{p}{q}\tan\varphi. \tag{69}$$

Equation (69) can be used to determine the following: If the critical angle for the topological phase transition of the chains is given by φ_0, then the positions of the eight Majoranas are given by the eight conditions

$$\begin{aligned} \tan\theta &= \frac{q}{p}\tan(\varphi \pm \varphi_0), \qquad \tan\theta = \frac{q}{p}\tan(\varphi + \pi \pm \varphi_0), \\ \tan\gamma &= \frac{q}{p}\tan(\varphi \pm \varphi_0), \qquad \tan\gamma = \frac{q}{p}\tan(\varphi + \pi \pm \varphi_0). \end{aligned} \tag{70}$$

Here, φ is the angle of the external magnetic field with the x_1-axis. Any pair of the 4×4 combinations of Majorana positions θ and γ from Eq. (70) can be viewed as a parametric curve in the θ-γ-plane, parametrized by $\varphi \in [0, 2\pi]$. These parametric curves are given for the case of two circles and a specific arrangement of two ellipses in Supplementary Figure 11(d) and (f), respectively.

To see the relevance of this plot for braiding operations, let us momentarily consider the unphysical case of two circles, with one Majorana state on each circle, as depicted in Supplementary Figure 11(a). The relative arrangement of the two circles is determined by the interval of the angles θ and γ that is enclosed by the other circle [red and orange segment in Supplementary Figure 11(a)]. In Supplementary Figure 11(b), this translates into a red and orange region that have an intersection spanned by the black line. Whether the two Majorana states braid upon a 2π field rotation can now be determined as follows. If the parametric curve that describes their position in the θ-γ plane as the field is rotated [the gray curve labeled i) in Supplementary Figure 11(b)] does not intersect the black line, the Majoranas do not braid. If the parametric curve that describes their position in the θ-γ plane as the field is rotated intersects the black line [gray curve labeled ii) in Supplementary Figure 11(b)], the Majoranas braid.

We shall now consider the physical case of four Majorana states on each circle [i.e., an ellipse with $p = q$, see Supplementary Figure 11(c)]. Before returning to the more general case of two ellipses with four Majoranas each, we will convince ourselves that any arrangement of two circles is too symmetric to perform a σ_x gate operation. The trajectories of each pairing of a Majorana from the first circle with a Majorana from the second circle are shown as gray lines in Supplementary Figure 11(d). For the case of perfect circles, these are straight lines with slope 1. As we shall see below, to have a pair of qubits that each performs a σ_x gate operation, it is required

that two times an odd number of parametric curves intersects the black line in Supplementary Figure 11(d). However, the red and orange intersection intervals cannot be chosen independently. Rather the center γ' of the orange interval and the center θ' of the red interval are related by $\gamma' + \pi = \theta'$. One verifies that in this case the black line in Supplementary Figure 11(d) always embraces two times an *even* number of gray curves, as these curves are arranged symmetrically around the curve defined by $\gamma + \pi = \theta$ (Notice that several parametric curves fall on top of one another in Supplementary Figure 11(d) and consult the figure caption for the correct counting of these degeneracies). This statement is independent of the critical angle φ_0. We conclude that two circles cannot be used to perform the desired braiding operation for a σ_x gate.

Finally, let us consider the case of two ellipses with $p \neq q$ and take for concreteness the values $p = 2q$ as well as the relative arrangement shown in Supplementary Figure 11(e). We label the Majorana states on one ellipse a_1, b_1, c_1 and d_1 and on the other ellipse a_2, b_2, c_2 and d_2. For the ellipses, the parametric curves shown in Supplementary Figure 11(f) are not straight lines and the black line that characterizes the intersection of the ellipses can embrace an odd number of them. Again, notice that several parametric curves fall on top of one another in Supplementary Figure 11(f). The black line is crossed by a 4-fold degenerate curve that corresponds to the Majorana pairs a_1-c_2, a_2-c_1, b_1-d_2 and b_2-$d_1$as well as by annother 2-fold degenerate curve is the trajectory of the pairs a_1-b_2 and c_1-d_2, making a total of 2×3 trajectories crossing it. Altogether, we obtain the number of pairwise braidings upon a 2π rotation of the magnetic field as summarized in Supplementary Table 1. We note that each Majorana in either ellipse also braids around all the other Majoranas in this ellipse. The last column of Supplementary Table 1 denotes the parity of the braiding operation on the respective Majorana, that is, the sign that the respective Majorana operator acquires.

We can now pair up the neighboring Majoranas and form a qubit from a_1, b_1 together with a_2, b_2 and a second qubit from c_1, d_1 together with c_2, d_2 in the way explained in Eq. (64). Each of these pairs of Majoranas acquires a relative phase of -1 during the 2π field rotation, resulting in a change of parity of the pair $P_{a_i b_i} \to -P_{a_i b_i}$, $P_{c_i d_i} \to -P_{c_i d_i}$, $i = 1, 2$. Hence, each of the two qubits changes its state from $|\bar{0}\rangle$ to $|\bar{1}\rangle$ and vice versa – the σ_x gate operation.

B. Braiding matrices and many-body Berry phases from simulations

In the previous section we derived the transformations of the states of the qubits solely based on the topology of their paths during the braid. In this section, we analyze the actual adiabatic evolution of the system under the field rotation. To that end, we reduce our original system to a simplified model in which we can extract the overall many-body Berry phase acquired by the ground states, together with the monodromy braiding matrix, upon braiding Majoranas. The simplified models are based on a small subset of a lattice which realizes two-dimensional spinless p-wave superconductivity. To be specific, such a system is described by the following Hamiltonian

$$\begin{aligned} H = &\sum_{\mathbf{n}=(n_x,n_y)} \frac{1}{2}\left[\mathbf{c}_\mathbf{n}^\dagger(i\Delta_{\mathbf{n},x}\sigma_x - t_{\mathbf{n},x}\sigma_z)\mathbf{c}_{\mathbf{n}+(1,0)} + \mathbf{c}_\mathbf{n}^\dagger(-i\Delta_{\mathbf{n},y}\sigma_y - t_{\mathbf{n},y}\sigma_z)\mathbf{c}_{\mathbf{n}+(0,1)} + \text{h.c.}\right] \\ &- \sum_{\mathbf{n}=(n_x,n_y)} \mu_\mathbf{n}\mathbf{c}_\mathbf{n}^\dagger\,\sigma_z\,\mathbf{c}_\mathbf{n}, \end{aligned} \tag{71}$$

where $\mathbf{c}_\mathbf{n} = (c_\mathbf{n},\ c_\mathbf{n}^\dagger)^T$ and all $\mu_\mathbf{n}$, $\Delta_{\mathbf{n},x/y}$ and $t_{\mathbf{n},x/y}$ are real. It is possible to base our calculation on this simplified model, as the spinless p-wave superconductor is a topologically equivalent effective description of our original system.

The operators $c_\mathbf{n}^\dagger$ that create spinless fermions are only defined on a subset of the sites of the lattice on which the p-wave superconductor resides. The panels (a) and (b) of Supplementary Figure 12 show two different choices of this subset, which represent a single ring and two linked rings, respectively. In each case we will abandon all the sites and bonds that are not highlighted. For simplicity, we also set $\Delta_{\mathbf{n},x} = t_{\mathbf{n},x}$ and $\Delta_{\mathbf{n},y} = t_{\mathbf{n},y}$ for each link of neighboring sites that remains. For each of these two models, we now vary slowly the set of parameters $\mathbf{p}(\lambda)$, with λ from 0 to 1 and $\mathbf{p}(0) = \mathbf{p}(1)$, to simulate the desired operation on the Majoranas. Here, $\mathbf{p}$ represents a vector that contains all the couplings $\mu_\mathbf{n}$, $t_{\mathbf{n},x}$, and $t_{\mathbf{n},y}$ of the model. The braiding matrix associated with a specific trajectory in parameter space and a specific qubit choice can be computed numerically. By dividing the range of λ into sufficiently small segments of length δ_λ, the unitary transformation that represents the braiding operation in the topologically degenerate ground state manifold is well approximated by

$$M_{ij} = \langle GS^{(i)}_{\lambda=1}|P_{\lambda=1-\delta_\lambda}P_{\lambda=1-2\delta_\lambda}\cdots P_{\lambda=\delta_\lambda}|GS^{(j)}_{\lambda=0}\rangle, \tag{72}$$

if the step δ_λ is small enough. Here, $GS^{(i)}_\lambda$ is the i-th ground state (with respect to a specific qubit

choice) for $\mathbf{p}(\lambda)$, $GS^{(i)}_{\lambda=1} = GS^{(i)}_{\lambda=0}$ for all i, and $P_\lambda = \sum_i |GS^{(i)}_\lambda\rangle\langle GS^{(i)}_\lambda|$ is the projector to the ground state subspace at λ.

Let us first consider the single-ring case. With the labeling indicated in Supplementary Figure 12(a), one can verify, for example, when $\mu_3 = \mu_4 = t_{1x} = t_{5x} = 1$ and all other parameters are zero, that there are four Majorana states, labeled by a, b, c and d, localized at sites 2, 1, 5 and 6, respectively. Varying the parameters adiabatically according to linear interpolations between the parameter values listed in Supplementary Table 2 leads to exchanges of a and c, and b and d. By choosing the qubits according to two fermionic operators

$$f_1 = \gamma_c + i\gamma_a,\ f_2 = \gamma_b + i\gamma_d, \tag{73}$$

where $\gamma_{a,b,c,d}$ stand for the Majorana operators defined at time 0 for the four Majorana states, we find the braiding matrix contains only diagonal elements that correspond to the mapping

$$\begin{aligned}&|00\rangle \to e^{i\varphi}|00\rangle,\ |11\rangle \to -e^{i\varphi}|11\rangle,\\&|01\rangle \to -ie^{i\varphi}|01\rangle,\ |10\rangle \to -ie^{i\varphi}|10\rangle,\end{aligned} \tag{74}$$

under the braiding operation. Here the global phase factor $e^{i\varphi}$ contains the process-dependent many-body Berry phase. For the specific operation taken above, $e^{i\varphi} \approx e^{i0.80\pi}$; if we insert another intermediate stage between stages 1 and 2, for example, characterized by $\mu_1 = \mu_2 = \mu_5 = \mu_6 = t_{1x} = t_{5x} = t_{2y} = t_{3y} = 1$ and otherwise 0, which only alters the specific trajectory of the parameters when linearly interpolated, but does not change the topology of the braiding operation, we find $e^{i\varphi} \approx e^{i0.83\pi}$.

The simple and explicit model here also allows us to estimate certain kinds of errors in the operation. For instance, the parameters that we have chosen so far have an ideal property that all Majorana states are decoupled (staying at exactly zero energy) at all time during the operation. By allowing the parameters that are set to zero to be finite but small, the Majorana states become coupled and this coupling will lead to errors in a qubit operation. Although theoretically such coupling among Majorana states can be exponentially small when their separations are large enough, for real experiments it is certainly important to estimate this kind of errors. For simplicity, we set all parameters that were previously set to 0, according to Supplementary Table 2, to the same small value ϵ, and investigate how the errors vary with ϵ. Here, ϵ physically represents the energy scale of the coupling among the Majorana states. In terms of the operation we have discussed on the single ring, we consider two quantities for the errors: the magnitude δ of the off-diagonal elements

in the braiding matrices, and the phase difference $\delta\varphi$ between the diagonal elements (excluding the expected relative sign change for states $|00\rangle$ and $|11\rangle$). These errors, for both parity sectors, are shown with respect to ϵ in Supplementary Figure 13.

We now turn to the case of two linked circles. With the labeling indicated in Supplementary Figure 12(b), one can check that when $\mu_{A3} = \mu_{A4} = \mu_{B3} = \mu_{B4} = t_{A1x} = t_{A4x} = t_{A5x} = t_{B1x} = t_{B3x} = t_{B5x} = 1$ and all other parameters are zero, there are in total eight Majorana states, labeled by $a_{1,2}$, $b_{1,2}$, $c_{1,2}$ and $d_{1,2}$ localized at sites $A2/B2$, $A1/B1$, $A5/B5$ and $A6/B6$, respectively. By varying the parameters according to linear interpolations between the parameter settings listed in Supplementary Table 3, one realizes a Majorana braiding operation equivalent to Supplementary Table 1. By choosing the qubits according to four fermionic operators

$$f_1 = \gamma_{b_1} + i\gamma_{a_1},\ f_2 = \gamma_{c_1} + i\gamma_{d_1},\ f_3 = \gamma_{c_2} + i\gamma_{d_2},\ f_4 = \gamma_{b_2} + i\gamma_{a_2}, \tag{75}$$

where the Majorana operators γ are defined at time 0, we find the braiding matrix is skew-diagonal which implies

$$|f_4 f_3 f_2 f_1\rangle \to e^{i\varphi}|\overline{f_4 f_3 f_2 f_1}\rangle \tag{76}$$

under the operation. Here, the overhead bar means a flip of all qubits (i.e., changing the fermionic occupation 0 and 1), and the (global) many-body Berry phase factor is obtained as $e^{i\varphi} \approx e^{i0.42\pi}$ for this specific operation. This confirms that the braiding operation indeed implements two copies of a σ_x gate, as claimed in the previous section.

Similar to the single-ring case, we can estimate the errors incurred in the current operation when the couplings among Majorana states are taken into account. Again for simplicity, we set all parameters that were previously set to 0, according to Supplementary Table 3, to the same small value ϵ, and investigate how the errors vary with ϵ. Here, we define two quantities for the errors: δ is the maximum deviation from 1, of the abstract values of the skew-diagonal elements in the braiding matrix; $\delta\varphi$ is the maximum deviation of the phases of the skew-diagonal elements from the common (global) phase. We find that (see Supplementary Figure 14), whereas the probability error δ increases fast beyond some threshold ($\epsilon \sim 10^{-6}$ in this case), the phase error $\delta\varphi$ is surprisingly almost independent on ϵ.

C. A trijunction to implement a braid between two Majorana bound states

A braiding operation between two Majorana states does not change the parity of a two-Majorana qubit. To keep matters simple, we can thus demonstrate it using a single pair of Majorana fermions. The goal is to braid the two Majorana bound states in a nearly adiabatic operation. This can be achieved using a trijunction of three linear chains. If the critical angle $\varphi_0 \in [\pi/6, \pi/3]$, either one or two of the three chains are in a topological state for each orientation of the magnetic field $\mathbf{B}$ (see Supplementary Figure 15). Note that φ_0 can be conveniently tuned to the desired value by changing $|\mathbf{B}|$. Then, for each field orientation there are precisely 2 Majorana bound states in the trijunction. Figure. 15 illustrates how a 2π rotation of the magnetic field in the 1-2 plane precisely implements a braid of the two Majorana states.

D. Rotating magnetic field from a combination of current pulses

As outlined in the main text, it is a technological challenge to achieve a rotating magnetic field with high enough frequency of rotation to perform this braiding operation. We propose to use current pulses of crossed conducing channels to achieve this, as outlined in Supplementary Figure 16.

Supplementary References

[1] S. Nadj-Perge, I.K. Drozdov, B.A. Bernevig, and A. Yazdani, Phys. Rev. B **88**, 020407(R) (2013).

[2] Y. Kim, M. Cheng, B. Bauer, R. M. Lutchyn, and S. Das Sarma, Phys. Rev. B **90**, 060401(R) (2014).
